# Public Maternal Health Dashboards in the United States: Descriptive Assessment

**DOI:** 10.2196/56804

**Published:** 2024-09-17

**Authors:** Jennifer A Callaghan-Koru, Paige Newman Chargois, Tanvangi Tiwari, Clare C Brown, William Greenfield, Güneş Koru

**Affiliations:** 1 Fay W Boozman College of Public Health University of Arkansas for Medical Sciences Springdale, AR United States; 2 Department of Obstetrics & Gynecology University of Arkansas for Medical Sciences Little Rock, AR United States; 3 Fay W Boozman College of Public Health University of Arkansas for Medical Sciences Little Rock, AR United States; 4 Family Health Branch Arkansas Department of Health Little Rock, AR United States

**Keywords:** dashboard, maternal health, data visualization, data communication, perinatal health

## Abstract

**Background:**

Data dashboards have become more widely used for the public communication of health-related data, including in maternal health.

**Objective:**

We aimed to evaluate the content and features of existing publicly available maternal health dashboards in the United States.

**Methods:**

Through systematic searches, we identified 80 publicly available, interactive dashboards presenting US maternal health data. We abstracted and descriptively analyzed the technical features and content of identified dashboards across four areas: (1) scope and origins, (2) technical capabilities, (3) data sources and indicators, and (4) disaggregation capabilities. Where present, we abstracted and qualitatively analyzed dashboard text describing the purpose and intended audience.

**Results:**

Most reviewed dashboards reported state-level data (58/80, 72%) and were hosted on a state health department website (48/80, 60%). Most dashboards reported data from only 1 (33/80, 41%) or 2 (23/80, 29%) data sources. Key indicators, such as the maternal mortality rate (10/80, 12%) and severe maternal morbidity rate (12/80, 15%), were absent from most dashboards. Included dashboards used a range of data visualizations, and most allowed some disaggregation by time (65/80, 81%), geography (65/80, 81%), and race or ethnicity (55/80, 69%). Among dashboards that identified their audience (30/80, 38%), legislators or policy makers and public health agencies or organizations were the most common audiences.

**Conclusions:**

While maternal health dashboards have proliferated, their designs and features are not standard. This assessment of maternal health dashboards in the United States found substantial variation among dashboards, including inconsistent data sources, health indicators, and disaggregation capabilities. Opportunities to strengthen dashboards include integrating a greater number of data sources, increasing disaggregation capabilities, and considering end-user needs in dashboard design.

## Introduction

### Background

Data dashboards are “visual displays that feature the most important information needed to achieve specific goals captured on a single screen” [1]. The term dashboard is borrowed from the vehicle dashboard, highlighting the expectation that a data dashboard will display key indicators used to understand performance and make decisions. Data dashboards can be designed to meet different types of goals, including informing strategic decisions, monitoring organizational performance, and communicating information to raise awareness and motivation [1-3]. Similar to businesses, health care organizations and public health agencies have widely adopted dashboards to support internal operations [4,5]. More recently, dashboards are being developed for the public communication of health-related data, a practice that accelerated during the COVID-19 pandemic [6,7]. Examples of public health dashboards include the City Health Dashboard [8], national and state substance use and overdose dashboards [9-11], and various COVID-19 dashboards [12,13].

The interest in public health dashboards has also expanded to the area of maternal health. The status of maternal health in the United States has been characterized as a crisis [14], with outcomes much worse than those in peer nations as well as large racial and ethnic disparities across perinatal outcomes [15,16]. National plans for improving maternal health have highlighted the need for better, more timely maternal health data [14,15]. Public reporting of maternal health data has also been proposed as a key mechanism for enabling quality improvement and empowering patient decision-making [17]. Consistent with these priorities, recent federal investments in maternal health have supported improving data systems. Several federal agencies are partnering on maternal data infrastructure projects to standardize measurement and expand surveillance to include underreported conditions and outcomes across settings [18]. At the state level, the Centers for Disease Control and Prevention (CDC) granted 39 awards to support maternal mortality review committees (MMRCs) in the collection and reporting of data on the causes of maternal deaths [19]. In the same year, the Health Resources and Services Administration awarded 9 new state maternal health innovation awards, with an emphasis on maternal health data. One of the primary suggested data-related innovations is the development of a state-focused maternal health dashboard “to easily access and report on maternal health outcomes” [20].

Maternal health data in the United States currently comes from multiple sources that are typically disconnected outside of limited data linkage efforts [18,21]. These include maternal mortality reviews conducted by state committees [22], national and state vital statistics systems [15], hospital discharge data [23], claims data [24], and surveys such as the Pregnancy Risk Assessment Monitoring System (PRAMS) [25]. Additional sources of health system, demographic, and social data may also identify risks and opportunities for improving maternal health. Maternal health dashboards have the potential to reduce access barriers to these data, particularly when integrating multiple sources of information in 1 location. Dashboards may also support efforts to address maternal health disparities through disaggregation and comparison features [26].

Alongside the growth of public health dashboards, questions have been raised regarding the extent to which dashboards present the most valuable data in a way that meets users’ information needs [4,7,13]. Published assessments of dashboards in other health areas have identified substantial heterogeneity and gaps in their actionability [7,27]. Segmenting data presentations for different audiences is considered essential for the utility of data dashboards, as audiences may differ in their information needs as well as their health and data literacy levels [3,4]. However, dashboard developers are often disconnected from public users and may have a limited understanding of users’ needs and abilities [4]. Given that there are few published reports on the development process for maternal health dashboards [28,29], little is known about the factors considered in maternal health dashboard design.

### This Study

To inform the development of a maternal health dashboard for the state of Arkansas, we undertook a rigorous descriptive assessment of existing publicly available maternal health dashboards in the United States. This study, the first step in a user-centered design process, allowed the team to identify the range of data sources and technical features in existing dashboards of similar scope. This paper provides the results of our evaluation of the content and features of existing publicly available maternal health dashboards in the United States. Given the strong interest in improving access to maternal health data [17] and the importance of maintaining and continually improving current dashboards [6], the results of this review of public dashboards are relevant to all stakeholders concerned with the dissemination of maternal health data.

## Methods

### Overview

This descriptive assessment was conducted by a team of researchers with expertise in information systems, software engineering, maternal health surveillance, and quantitative and qualitative methods. Similar to scoping reviews of published literature [30], we sought to identify the scope and key attributes of public dashboards presenting maternal health data for the United States. The methods used for this assessment were also informed by prior studies of hospital patient safety dashboards [27] and public COVID-19 dashboards [7,31].

### Identification of Dashboards

Between January and March 2023, the research team performed multiple searches of publicly available maternal health dashboards using the Google (Google LLC) search engine. The topical keywords included in each search were “dashboard,” “maternal,” “perinatal,” and “birth.” Given the geographic focus of the study, each search contained a geographic keyword, either “United States” or the name of 1 of the 50 states (eg, Alabama, Alaska, and Arizona). A total of 51 separate searches were performed, and each search was completed independently by 2 team members. Team members accessed and reviewed the first 10 results for each search they performed to determine whether the websites met the eligibility criteria.

We adopted the functional definition of a dashboard from the study by Sarikaya et al [3], “an interactive display that enables...monitoring of dynamically updating data,” as the basis for our inclusion criteria. A dashboard was eligible for inclusion if it (1) reported data related to maternal health, (2) included data from the United States, (3) was accessible by the public, and (4) included at least 1 interactive feature for data presentation. A dashboard was considered ineligible if it (1) was presented on a password-protected, nonpublic website; (2) provided data only as downloadable files (eg, PDF reports and Excel spreadsheets); (3) did not contain any interactive features; (4) did not include any US data. All dashboards determined to be eligible by the team were included in a list for data extraction. Through the searches, 76 websites were identified. An additional 4 dashboards were identified during the review of the content of included dashboards and state health department websites and were added to the abstraction list, resulting in a total of 80 dashboards (Multimedia Appendix 1).

### Abstraction of Dashboard Characteristics

The features and content of identified dashboards were abstracted using a standardized form in REDCap (Research Electronic Data Capture; Vanderbilt University). After a preliminary review of the first quarter of identified dashboards, the team developed an abstraction form to capture the variability in features and data observed across this subset of dashboards. Following the initial abstraction of all dashboards, the form underwent a revision to explicitly include dashboard features and content that were most commonly included in “other” categories, as described subsequently. The final form included >250 fields across four areas: (1) scope and origins, (2) technical capabilities, (3) data sources and indicators, and (4) disaggregation capabilities (Multimedia Appendix 2).

“Scope and origins” fields addressed the geographic areas and health topics included in the dashboard, the dashboard’s stated purpose and audience, and the hosting organization or website. “Technical capabilities” fields included types of visualizations, export and download features, responsiveness and mobile friendliness, and available comparisons (eg, longitudinal trends and benchmarks). Both the “scope and origins” and “technical capabilities” fields were assessed for the website as a whole, except for visualizations, which were abstracted only for maternal health data. “Data sources and indicators” fields addressed the maternal health data sources explicitly reported on the dashboard as well as the presence, period, and format of specific maternal or perinatal indicators. A total of 40 indicators were abstracted into 4 categories: health status or outcomes (n=11, 28% indicators), health behaviors and use of health services (n=11, 28% indicators), individual characteristics and risk factors (n=12, 30% indicators), and health system characteristics (n=5, 12% indicators). “Disaggregation capabilities,” also assessed for maternal health data, included fields for abstracting disaggregation by race, geography, and multiple perinatal characteristics. A variable that could be used for disaggregation was considered to additionally be an indicator on the dashboard only if the variable was also presented independently of disaggregation functions. Most fields on the abstraction form were assessed as binary variables, indicating the presence or absence of a data point or feature. “Other” options were included for each section on the abstraction form, with the text specification of the variable or feature observed. A small set of fields, related to the stated purpose and audience of each dashboard, also required the abstraction of textual information. Operational definitions were developed by the team for select abstraction fields that required interpretation by abstractors.

The original abstraction process was completed by 5 team members between February and May 2023. A total of 2 (40%) master’s-trained team members were responsible for the primary abstraction of all dashboards. A total of 3 (60%) PhD-trained team members, each with at least 5 years of experience working with US maternal health data, completed a thorough secondary review for each dashboard. All corrections made during the secondary review were logged by REDCap, with a note of explanation from the reviewer. Given that website features may change or websites may become unavailable at any time, 1 team member also recorded brief videos demonstrating the primary features of each website at the time of abstraction. A second round of abstraction for an additional 9 variables was completed in May 2024 to respond to reviewer comments. Of the 80 included dashboards, 5 (6%) were no longer accessible, and additional abstraction for these websites was completed to the extent possible using the recorded videos.

### Analysis

Following the completion of reviews, the abstracted data were exported from REDCap into R (version 4.3.1; The R Foundation) [32] for analysis. The number and proportion of dashboards with each characteristic were calculated. To characterize the stated purpose and audience, the team followed a qualitative content analysis approach, led by a PhD-trained team member with 15 years of qualitative and mixed methods research experience. The abstracted text that described purpose and audience was exported into a matrix and read for familiarization and the inductive identification of common themes. A total of 8 thematic categories were identified in the dashboard purpose descriptions, and 6 categories were identified in the dashboard audience descriptions. Operational definitions were developed for each of these 14 categories. After the REDCap form was modified to add these categories, 1 team member reviewed all definitions again to apply the relevant categories, and a second team member checked all purpose and abstract characterizations with reference to the abstracted text and original websites, as needed. Illustrative quotes for each purpose category were selected for presentation in the Results section.

### Ethical Considerations

This study collected data from publicly available websites. It did not include individual-level data, and did not involve any interaction with human subjects. Therefore, the study did not meet the definition of human subjects research according to the University of Arkansas for Medical Science’s “Human Subjects Protection Program Plan” (version 6**/**24**/**2021).

## Results

### Scope and Origins

Among the 80 publicly available maternal health dashboards included in this study, 24 (30%) were solely focused on maternal health; 28 (35%) included both maternal and child health data; and 28 (35%) included data for health areas outside of maternal and child health, such as environmental health and chronic diseases (Table 1). A total of 46 (58%) dashboards included the term “dashboard” in the website title or description, while 32 (40%) did not. Most dashboards (58/80, 72%) had a focus on state-level data, 8 (10%) presented national or multinational data, and 14 (18%) presented data for a substate region (eg, a city, multiple counties, or a tribal area). The most common hosting website for included dashboards was a health department website (48/80, 60%), followed by a nongovernmental organization or program website (14/80, 18%). Other types of hosting websites included those of federal agencies and universities (Multimedia Appendix 3). The software platforms used to create reviewed dashboards included Tableau (Tableau Software, LLC; 30/80, 38%); unspecified or custom systems (26/80, 32%); and off-the-shelf solutions (16/80, 20%), such as Conduent (Conduent Inc), Clear Impact (Clear Impact, LLC), MySidewalk (MySidewalk, Inc), and IBM Cognos (IBM Corp). A small number of dashboards were created with Power BI (Microsoft Corp; 4/80, 5%) or ArcGIS (Environmental Systems Research Institute, Inc; 4/80, 5%).

**Table 1:** Design and features of reviewed dashboards (N=80).

Characteristic^a^			Dashboards, n (%)
**Topical scope**			
	Maternal and child health	28 (35)	
	Broader than maternal and child health	28 (35)	
	Maternal health only	24 (30)	
**Geographic scope**			
	National or multinational	8 (10)	
	State focus	58 (72)	
	Region or other area	14 (18)	
**Use of the term “dashboard” in the title or description^b^**			
	Includes the term “dashboard”	46 (58)	
	Does not include the term “dashboard”	32 (40)	
**Hosting organization or website**			
	Health department web page	48 (60)	
	NGO^c^ or program web page	14 (18)	
	Other or unspecified	18 (22)	
**Visualizations of maternal health data**			
	Table	63 (79)	
	Bar chart	58 (72)	
	Line graph	52 (65)	
	Map	46 (58)	
	Pie chart	14 (18)	
	Large numbers or card visualizations	9 (11)	
	Arrows representing the direction of change	7 (9)	
	Gauge chart	5 (6)	
	Other visualization type	9 (11)	
	CIs in any visualizations	24 (30)	
**Software platform**			
	Tableau	30 (38)	
	Unspecified or custom system	26 (32)	
	Off-the-shelf solutions^d^	16 (20)	
	Power BI	4 (5)	
	ArcGIS	4 (5)	
**Technical capabilities**			
	User can download data	46 (58)	
	User can download complete dashboard view	43 (54)	
	All indicators visible on one page	38 (48)	
	Responsive interface	37 (46)	
	Instructions for interactive features	35 (44)	
	Mobile-friendly interface	31 (39)	
	User can download individual visualizations	26 (32)	
	User can select visualization type	16 (20)	
	Adaptive visualization (select to filter)	8 (10)	
	Full screen	6 (8)	
	Other	21 (26)	
**Statements of purpose and audience**			
	Statement of purpose	55 (69)	
	Statement of intended audience	30 (38)	
**Stated audience categories^e^**			
	Legislators or policy makers	17 (57)	
	Public health agencies or organizations	16 (53)	
	General audience descriptor	15 (50)	
	Health care organizations or providers	11 (37)	
	Community members or health care consumers	10 (33)	
	Researchers	7 (23)	
	Other	8 (27)	

^a^All characteristics were assessed for the dashboard as a whole, except for visualizations, which was restricted to maternal health data.

^b^Could not be assessed for 2 dashboards that were no longer accessible during the second round of abstraction.

^c^NGO: nongovernmental organization.

^d^Off-the-shelf systems include Conduent and MySidewalk.

^e^Percentages calculated among the subset of 30 dashboards with a stated audience.

### Technical Capabilities and Visualizations

Included dashboards used a range of data visualizations and technical features (Table 1). A total of 4 visualization types were used in most dashboards: tables (63/80, 79%); bar charts (58/80, 72%); line graphs (52/80, 65%); and maps (46/80, 58%), predominantly choropleth maps. Less common visualizations included pie charts (14/80, 18%), large numbers or card visualizations (9/80, 11%), directional arrows (7/80, 9%), and gauge charts (5/80, 6%). The most common technical features observed for included dashboards were the ability to download data (46/80, 58%) and the ability to download the dashboard view as an image or document file (eg, in PDF) (43/80, 54%). The classic dashboard style of presenting all indicators on 1 page was observed in roughly half of the reviewed dashboards (38/80, 48%). Some dashboards also accommodated different screen sizes with responsive interfaces (37/80, 46%) and mobile-friendly interfaces (31/80, 39%). Less common technical features included the option allowing the user to select the type of visualization (16/80, 20%), adaptive visualizations (8/80, 10%), and full-screen mode (6/80, 8%).

### Audience and Purpose

While most included dashboards provided an explicit statement regarding the purpose of the dashboard (55/80, 69%), descriptions of the intended audience were less common (30/80, 38%), with close to two-thirds (50/80, 62%) of dashboards not specifying their audience (Table 1). A total of 8 categories of audiences were identified from the dashboards’ textual statements regarding audience. Audience categories specified in at least half of the audience statements included legislators or policy makers (17/30, 57%); public health agencies or organizations (16/30, 53%); and general audience descriptors (15/30, 50%), such as “stakeholders,” “partners,” or general “public” audiences. Health care organizations or providers (11/30, 37%) and community members or health care consumers (10/30, 33%) were identified in around one-third of the statements, while the least common category was researchers (7/30, 23%).

The thematic analysis of dashboards’ purpose statements identified 8 distinct categories of purpose: data accessibility, program planning or needs assessment, accountability for government organizations, program evaluation and monitoring, research, quality improvement, policy making and goal setting, and individual decision-making (Table 2). Among the 55 dashboards with a statement of purpose, general data accessibility was the most common purpose and was noted in 35 (64%) dashboards. Often, the dashboard was described as a tool to achieve a public agency’s goals or mandates for data accessibility. The second most common purpose was program planning or needs assessment (n=24, 44%), which included identifying health needs, developing plans to address these needs, and applying for funding to support program plans. Accountability for government-supported programs (eg, Medicaid) and health care organizations was included in some purpose statements (n=14, 25%), as was program evaluation and monitoring (n=14, 25%). The least common purpose categories were research (n=8, 15%), quality improvement for health care organizations (n=7, 13%), and policy making and goal setting (n=4, 7%). Supporting individual decision-making by health care consumers or community members, such as selecting a health care provider or identifying a healthy behavior, was observed in a small proportion of purpose statements (n=4, 7%). Other types of purposes included journalism and promoting residency or tourism in the area (Multimedia Appendix 3).

**Table 2:** Purpose categories and example statements among dashboards that provided a statement of purpose (n=55).

Purpose category	Values, n (%)	Example statements
Data accessibility	35 (64)	Idaho Vital Statistics Natality Dashboard [33]^a^: “The Division of Public Health’s Strategic Plan has identified priority areas which include leveraging and using data more effectively across the Division as well as improving data accessibility for use by stakeholders (i.e., public health agencies, health systems, decision-makers, and the public). The data found on this website are intended to help the Division of Public Health achieve those priority area strategic goals.”
Program planning and needs assessment	24 (44)	Georgia Online Analytical Statistical Information System (OASIS^b^) [34]: “OASIS plays an integral role in program planning, which includes determining target population areas, formulating financial plans, monitoring program effectiveness, program evaluation and reporting program outcomes. Use OASIS data querying tools to: develop profiles and report cards for counties or districts; assess community health needs, prioritize health problems, and evaluate programs; assemble data for grant writing, health analysis, special projects or state legislative reporting.”
Accountability for government and organizations	14 (25)	Missouri HealthNet Managed Care Quality Dashboard [35]: “The purpose of the Medicaid Managed Care Quality Dashboard is to provide transparency and accountability in the health care provided to Missouri’s Medicaid participants.”
Program evaluation and monitoring	14 (25)	Montana Children’s Health Data Dashboard [36]: “The Montana Children’s Health Data Dashboard highlights ten shared measures identified by early childhood health stakeholders. Stakeholders and early childhood coalitions can use these measures to track outcomes and demonstrate the impact of their work.”
Research	8 (15)	Hawai’i Health Data Warehouse [37]: “The Hawai’i Health Data Warehouse is dedicated to providing useful data to support public health professionals, researchers, the community and health agencies to become more effective in the application of health data.”
Quality improvement	7 (13)	Agency for Healthcare Research and Quality Perinatal Dashboard [38]: “This rich data source makes it possible to identify and track patient safety concerns for the purpose of learning how to mitigate patient safety risks and reduce harm across healthcare settings nationally.”
Policy making and goal setting	4 (7)	Kansas Health Matters [39]: “Kansas Health Matters is intended to help hospitals, health departments, community members and policy makers learn about the health of the community and ways to help improve it.... Master planners and government representatives can use this data to establish community goals on a variety of platforms.”
Individual decision-making	4 (7)	New Jersey Maternal Health Hospital Report Card [40]: “This is an informational resource tool that provides important data on maternal health care provided in New Jersey licensed birthing general acute care hospitals. In determining the best hospital for you, you may review the information provided in this report.”
Other	10 (18)	March of Dimes Peristats [41]: “Data are updated throughout the year, and useful for multiple tasks, including fact-finding,...lectures and presentations.”

^a^Reference numbers refer to the order of appearance in Multimedia Appendix 1.

^b^OASIS: Online Analytical Statistical Information System.

### Data Sources

Most dashboards reported data from only 1 (33/80, 41%) or 2 (23/80, 29%) sources (Table 3). Over half (47/80, 59%) of the dashboards reported state vital statistics as a data source. The second most common data source was the state PRAMS (22/80, 28%), followed by state health departments (12/80, 15%), the US Census (9/80, 11%), the CDC’s Wide-ranging Online Data for Epidemiologic Research system (9/80, 11%), and the National Center for Health Statistics (8/80, 10%). Data sources reported in <10% (8/80) of dashboards include hospital discharge data (6/80, 8%), the Health Resources and Services Administration (5/80, 6%), and insurance claims data (4/80, 5%). Of the 80 dashboards, 2 (2%) reported the state MMRC as a source of data. Narrative interpretations of the meaning of maternal health data were more commonly included on reviewed dashboards than narrative interpretations of data quality.

**Table 3:** Sources, comparisons, and disaggregation capabilities for maternal health data within reviewed dashboards (N=80).

Characteristic		Dashboards, n (%)
**Number of data sources reported**		
	1	33 (41)
	2	23 (29)
	3	5 (6)
	4	6 (8)
	≥5	10 (12)
	None stated	3 (4)
**Specified data sources**		
	State vital statistics	47 (59)
	PRAMS^a^	22 (28)
	State health department	12 (15)
	US Census	9 (11)
	CDC WONDER^b^	9 (11)
	National Center for Health Statistics	8 (10)
	Hospital discharge data	6 (8)
	Health Resources and Services Administration	5 (6)
	Insurance claims data	4 (5)
	Maternal mortality reviews	2 (2)
	Other^c^	31 (39)
**Narrative interpretations of data quality**		
	Completeness or validity	31 (39)
	Privacy protections	16 (20)
	Biases	2 (2)
**Narrative interpretations of meaning**		
	Definition of variable	51 (64)
	Normative interpretation of value	32 (40)
	Contextualization with factors influencing value^d^	52 (65)
**Comparisons**		
	Temporal	63 (79)
	Other geographic areas	42 (52)
	National data	25 (31)
	Benchmarks or targets	18 (22)

^a^PRAMS: Pregnancy Risk Assessment Monitoring System.

^b^CDC WONDER: Centers for Disease Control and Prevention Wide-ranging Online Data for Epidemiological Research.

^c^See Multimedia Appendix 3 for a description of other responses.

^d^Includes discussion of the influence of sample size on the reliability of estimates as well as policy or environmental influences.

### Comparisons and Disaggregation Capabilities

The comparison and data disaggregation capabilities also varied considerably between dashboards (Table 3). While a large majority of dashboards (63/80, 79%) provided temporal comparisons for included indicators, roughly half (42/80, 52%) allowed for comparisons between geographic areas. Some dashboards also provided comparisons against national data (25/80, 31%) and benchmark or target values (18/80, 22%), such as the Healthy People goals. The predominant period for reporting data was annual, followed by multiyear reporting periods (Multimedia Appendix 4).

Data disaggregation by race or ethnicity was possible for any data on 55 (69%) dashboards and for all data on 26 (32%) dashboards (Table 4). The next most common disaggregation variables for any data were maternal age (42/80, 52%) and insurance status or type (24/80, 30%). Few dashboards presented data by health care facility (7/80, 9%) or health care provider (5/80, 6%).

**Table 4:** Disaggregation capabilities of included dashboards (N=80).

Disaggregating variable	Dashboards, n (%)	
	Any data	All data
Time interval	65 (81)	54 (68)
Geography	65 (81)	53 (66)
Race or ethnicity	55 (69)	26 (32)
Maternal age	42 (52)	14 (18)
Insurance status or type	24 (30)	13 (16)
Education	18 (22)	5 (6)
Marital status	16 (20)	5 (6)
Receipt of social support	10 (12)	3 (4)
Birth weight	9 (11)	0 (0)
Level of poverty	7 (9)	3 (4)
Sex or gender of the mother or baby	7 (9)	2 (2)
Household income	7 (9)	2 (2)
Plurality	7 (9)	1 (1)
Health care facility	7 (9)	1 (1)
Nativity or citizenship	7 (9)	1 (1)
Gestational age	6 (8)	1 (1)
Health care provider	5 (6)	1 (1)
Other characteristics^a^	21 (26)	7 (9)

^a^See Multimedia Appendix 3 for a description of other responses.

### Indicators

Table 5 displays the presence of 40 specific perinatal health indicators in 4 groups, health status or outcomes, health behaviors and health care use, individual characteristics and risk factors, and health system characteristics, along with the format in which and the geographic level at which each is reported. Rates or percentages were more commonly used as the reporting format than counts for almost all health indicators. The most common geographic level for reporting health indicators was the state, followed by county and regions within states. Among health status indicators, birth weight (54/80, 68%), preterm birth (45/80, 56%), and infant mortality (42/80, 52%) were reported in at least half of the reviewed dashboards. Birth statistics were reported in 48% (38/80) of dashboards, and mode of delivery (eg, vaginal or cesarean delivery) was included in 26% (21/80) of dashboards. Indicators of any pregnancy-related problems, such as gestational diabetes or preeclampsia, were reported in 20% (16/80) of dashboards, while maternal mental health conditions were reported in 18% (14/80) of dashboards. Severe maternal morbidity and maternal mortality were each reported in <1 (20%) in 5 of the reviewed dashboards.

Among the 23 health indicators grouped under the “health behaviors and health care use” and “individual characteristics and risk factors” groups, only 3 (13%) were reported on half or more of the reviewed dashboards: receipt of prenatal care (57/80, 71%), maternal smoking (42/80, 52%), and maternal age (43/80, 54%). Indicators reported in 20% to 30% of dashboards include breastfeeding (22/80, 28%), preconception health (18/80, 22%), maternal BMI (17/80, 21%), health insurance status (17/80, 21%), and maternal education (17/80, 21%). The remaining 15 (65%) health indicators in the “health behaviors and health care use” and “individual characteristics and risk factors” groups were reported in <20% (16/80) of dashboards. Finally, “health system characteristics” was the least commonly included group of indicators. Among the 80 dashboards, indicators of population characteristics (eg, sex ratios, fertility rates, and pregnancy rates) and access to the Special Supplemental Nutrition Program for Women, Infants, and Children were reported in 18 (22%) and 16 (20%) dashboards, respectively. The availability of maternity care was reported in 12 (15%) dashboards, while policy measures and expenditures related to maternal health care were reported only in <5 (6%) dashboards.

**Table 5:** Maternal health indicators and reporting format among reviewed dashboards (N=80).

Category		Any indicator, n (%)	Format^a^, n (%)		Geographic level^a^, n (%)				
			Count	Percentage or rate	National	State	Substate region	County	Smaller than county
**Health status and outcomes**									
	Birth weight	54 (68)	27 (34)	50 (62)	15 (19)	48 (60)	20 (25)	37 (46)	7 (9)
	Preterm birth	45 (56)	20 (25)	41 (51)	11 (14)	42 (52)	17 (21)	30 (38)	3 (4)
	Infant mortality	42 (52)	19 (24)	39 (49)	16 (20)	35 (44)	13 (16)	31 (39)	3 (4)
	Births	38 (48)	32 (40)	23 (29)	7 (9)	30 (38)	13 (16)	30 (38)	7 (9)
	Mode of delivery	21 (26)	11 (14)	19 (24)	8 (10)	20 (25)	5 (6)	10 (12)	4 (5)
	Pregnancy-related problems	16 (20)	8 (10)	15 (19)	2 (2)	15 (19)	6 (8)	8 (10)	3 (4)
	Maternal depression or anxiety	14 (18)	5 (6)	14 (18)	2 (2)	14 (18)	6 (8)	4 (5)	1 (1)
	Severe maternal morbidity	12 (15)	5 (6)	9 (11)	4 (5)	12 (15)	3 (4)	2 (2)	1 (1)
	Maternal mortality	10 (12)	4 (5)	9 (11)	4 (5)	9 (11)	1 (1)	6 (8)	0 (0)
	Birth defects	9 (11)	5 (6)	9 (11)	2 (2)	6 (8)	1 (1)	4 (5)	0 (0)
	Neonatal abstinence syndrome	8 (10)	3 (4)	8 (10)	4 (5)	8 (10)	3 (4)	3 (4)	0 (0)
	Other	14 (18)	9 (11)	13 (16)	2 (2)	12 (15)	4 (5)	9 (11)	2 (2)
**Health behaviors and health care use**									
	Receipt of prenatal care	57 (71)	21 (26)	54 (68)	13 (16)	53 (66)	20 (25)	35 (44)	3 (4)
	Maternal smoking	42 (52)	16 (20)	41 (51)	10 (12)	39 (49)	16 (20)	22 (28)	0 (0)
	Breastfeeding	22 (28)	8 (10)	21 (26)	6 (8)	21 (26)	7 (9)	7 (9)	2 (2)
	Substance use	14 (18)	5 (6)	13 (16)	2 (2)	14 (18)	6 (8)	5 (6)	0 (0)
	Oral health	13 (16)	4 (5)	13 (16)	1 (1)	12 (15)	3 (4)	2 (2)	0 (0)
	Postpartum care	12 (15)	4 (5)	10 (12)	2 (2)	11 (14)	3 (4)	2 (2)	0 (0)
	Maternal nutrition	11 (14)	4 (5)	11 (14)	1 (1)	11 (14)	4 (5)	4 (5)	0 (0)
	Immunization	10 (12)	5 (6)	10 (12)	1 (1)	10 (12)	3 (4)	3 (4)	1 (1)
	Contraception	9 (11)	3 (4)	9 (11)	2 (2)	9 (11)	5 (6)	4 (5)	1 (1)
	Preventive health care visit	5 (6)	1 (1)	5 (6)	1 (1)	5 (6)	1 (1)	2 (2)	1 (1)
	Smoking household	5 (6)	2 (2)	5 (6)	1 (1)	5 (6)	3 (4)	3 (4)	2 (2)
	Other	19 (24)	9 (11)	17 (21)	3 (4)	18 (22)	7 (9)	8 (10)	3 (4)
**Individual characteristics and risk factors**									
	Maternal age	43 (54)	19 (24)	37 (46)	14 (18)	36 (45)	14 (18)	26 (32)	5 (6)
	Preconception health	18 (22)	8 (10)	17 (21)	1 (1)	16 (20)	5 (6)	6 (8)	3 (4)
	Maternal BMI	17 (21)	6 (8)	17 (21)	2 (2)	16 (20)	4 (5)	10 (12)	2 (2)
	Health insurance status	17 (21)	9 (11)	17 (21)	3 (4)	15 (19)	3 (4)	8 (10)	3 (4)
	Maternal education	17 (21)	11 (14)	14 (18)	4 (5)	16 (20)	5 (6)	11 (14)	3 (4)
	Pregnancy intention	13 (16)	5 (6)	13 (16)	2 (23)	13 (16)	5 (6)	3 (4)	0 (0)
	Stress or abuse	11 (14)	5 (6)	11 (14)	2 (2)	10 (12)	2 (2)	3 (4)	1 (1)
	Maternal marital status	10 (12)	9 (11)	8 (10)	1 (1)	10 (12)	4 (5)	9 (11)	1 (1)
	Plurality	9 (11)	7 (9)	8 (10)	1 (1)	9 (11)	3 (4)	8 (10)	2 (2)
	Birth spacing	7 (9)	6 (8)	6 (8)	1 (1)	6 (8)	3 (4)	6 (8)	2 (2)
	Number of prior births	7 (9)	5 (6)	7 (9)	0 (0)	7 (9)	3 (4)	4 (5)	1 (1)
	Maternal race or ethnicity	5 (6)	2 (2)	4 (5)	0 (0)	5 (6)	2 (2)	1 (1)	1 (1)
	Other	2 (2)	1 (1)	2 (2)	1 (1)	1 (1)	0 (0)	1 (1)	0 (0)
**Health system characteristics**									
	Population characteristic	18 (22)	7 (9)	17 (21)	3 (4)	16 (20)	5 (6)	13 (16)	4 (5)
	WIC access^b^	16 (20)	4 (5)	16 (20)	3 (4)	11 (14)	3 (4)	9 (11)	0 (0)
	Availability of maternity care	12 (15)	6 (8)	6 (8)	2 (2)	9 (11)	3 (4)	8 (10)	3 (4)
	Policy measures	4 (5)	3 (4)	1 (1)	3 (4)	2 (2)	2 (2)	1 (1)	0 (0)
	Health care expenditure	2 (2)	2 (2)	0 (0)	0 (0)	2 (2)	1 (1)	2 (2)	1 (1)
	Other	3 (4)	1 (1)	2 (2)	0 (0)	3 (4)	3 (4)	2 (2)	1 (1)

^a^Dashboards may use >1 reporting format and >1 geographic level.

^b^WIC access: Special Supplemental Nutrition Program for Women, Infants, and Children access.

## Discussion

### Principal Findings

This study demonstrates that public dashboards presenting US maternal health data are widespread. While most reviewed dashboards are hosted by state or local health departments, the content and features across maternal health dashboards are not uniform. Reviewed dashboards were developed with various software platforms and offered diverse technical capabilities. Only half (38/80, 48%) of the maternal health dashboard designs reflected the traditional, single-view presentation of key metrics, demonstrating the expanding conceptualization of dashboards [3]. The included dashboards also presented a range of maternal health indicators, generally reflective of their reported scope and included data sources. The heterogeneity in the design and content of maternal health dashboards is similar to that observed in public COVID-19 dashboards [7,31].

In addition to mapping the breadth of options for US maternal health dashboards, this study also suggests opportunities for improving the utility of current and future dashboards. Greater integration of multiple data sources is the first opportunity for improvement suggested by these results. Overall, 70% (56/80) of included dashboards in this study provide data from only 1 or 2 sources, yet individual data sources provide an incomplete picture of maternal health in the United States. Birth records, the primary vital statistics data reported on dashboards, exclude key outcomes, notably maternal mortality rates. It is particularly striking that only 2 (2%) of the 80 dashboards included data from MMRCs, the gold standard assessment of state-level maternal mortality [42]. Similarly, hospital discharge data files, the primary data source for calculating severe maternal mortality rates using the CDC’s algorithm [23], were a reported data source in only 6 (8%) of the 80 dashboards. State-focused dashboards, in particular, could enable more efficient access to maternal health data by including key maternal indicators from all state data sources—vital statistics (including death certificates), PRAMS, MMRCs, and hospital discharge data files—in 1 comprehensive dashboard.

A second area where public maternal health dashboards can be strengthened is their ability to identify disparities through data disaggregation. Maternal health disparities in the United States are of grave concern, with Black women and American Indian or Alaska Native women having 2 to 3 times greater risk of maternal death compared to White non-Hispanic women [14] and increased likelihood of receiving poor quality care [43]. Women residing in rural areas [44] and those with public insurance [43] also experience worse maternal outcomes. The ability of data systems to detect these disparities is considered critical for efforts aimed at reducing adverse perinatal outcomes overall as well as disparities in these outcomes [43,45,46]. While most of the reviewed dashboards permitted the disaggregation of all indicators by geography, only one-third (26/80, 32%) had the capability of disaggregation by race or ethnicity for all indicators. Disaggregation by other social determinants of maternal health, such as insurance type, education, and language spoken, was even less common. Although privacy protections may prohibit the stratified presentation of infrequent outcomes [47], such as maternal mortality, most individual-level indicators reported on reviewed dashboards would not require such data suppression.

Third, maternal dashboards may increase attention to the information needs of their audiences and segmentation of information presented for different audiences. Fewer than half (30/80, 38%) of the dashboards in this study provided statements about their intended audience; available audience statements typically listed multiple, diverse stakeholders. Critical assessments of public health dashboards have noted that “not all data are necessary for all users at all times, and often the information that citizens need to make informed decisions is absent” [4]. The interest and needs of policy makers and providers for publicly reportable maternal health data are not well studied. For maternity patients, documented information needs include identifying geographically close health care providers who provide the services and quality that the patient prefers [48-50]. These patient needs appear to be particularly underserved by current maternal health dashboards. Among reviewed dashboards, <1 (10%) in 10 presented any data by health care facility. The technical features and visual displays of dashboards should also be differentiated for different audiences [51]; while program planners and researchers may prioritize access to a wide range of variables available through sophisticated queries, maternity patient decision-making has been shown to benefit from explanations about the relevance of included data [52]. Engaging intended audience members during dashboard design and development is a best practice that was followed in 1 (50%) [29] of the 2 reports of public maternal dashboard development [28,29]. Future qualitative research with maternal health dashboard development teams, similar to COVID-19 dashboard studies [53], could lead to a better understanding of the factors guiding their decisions and opportunities to improve processes and dashboard actionability for intended users.

### Limitations

This study could not evaluate the impact of maternal health dashboards and was limited in the extent to which it could assess aspects of the user experience, such as usability. Although comprehensive usability standards have recently been proposed for public health data dashboards [54], further operationalization is needed before they can be consistently applied. The features of some dashboards suggest limited usability for many users. For example, fewer than half (31/80, 39%) of dashboards had a responsive interface that would reformat for viewing on a mobile device. This study was also unable to address the usefulness of the data and features of maternal health dashboards from the perspective of intended users. We could not identify any published articles evaluating the user experience, interaction effectiveness, system efficacy [55], or actionability [13] of a deployed maternal health dashboard, although these evaluations are critical to understanding and optimizing the contribution of public health dashboards to patient- and system-level decision-making [6].

### Further Considerations

For maternal health dashboards to successfully improve in sophistication and meet the expectations of multiple stakeholders, prior research suggests that it is important to involve collaborative interdisciplinary teams in dashboard design and development [53]. The expertise of systems analysts, software engineers specializing in backend and front-end web development, and user-interface designers is likely to result in dashboards with improved usefulness and usability. These fields have established best practices for designing usable systems that are often neglected in health information systems [56]. When resources are limited, general-purpose data analytics tools such as Tableau or Microsoft Power BI are promising low-cost options for prototyping. Ongoing data improvement efforts by national funders and organizations may also contribute resources and standards to support the efficient development of maternal data dashboards. For example, data standards could facilitate sharing, reuse, and integration of maternal health data [57], and user-interface standards could lead to more effective and efficient development by offering specific widgets and visualization solutions for certain maternal health data or for certain purposes (eg, benchmarking).

### Conclusions

This national descriptive assessment of maternal health dashboards in the United States found substantial variation among dashboards, including inconsistent data sources, health indicators, and disaggregation capabilities. Few dashboards included information regarding maternity patients as an intended dashboard audience. Careful consideration of the design of publicly available health dashboards, with specific intent to develop dashboards that cater to end-user needs and to include data from multiple sources, is critical for ensuring that dashboards achieve their intended goals.

## Appendix

Multimedia Appendix 1 List of reviewed maternal health dashboards.

Multimedia Appendix 2 REDCap (Research Electronic Data Capture) data abstraction instrument.

Multimedia Appendix 3 Description of other categories for Tables 1, 3, 4, and 5.

Multimedia Appendix 4 Indicator reporting periods.

## Abbreviations

CDC: Centers for Disease Control and Prevention
MMRC: maternal mortality review committee
PRAMS: Pregnancy Risk Assessment Monitoring System
REDCap: Research Electronic Data Capture
