# Supplementary material for: Public Maternal Health Dashboards in the United States: Descriptive Assessment
Source: J Med Internet Res. 2024 Sep 17;26:e56804. doi: 10.2196/56804 (PMC11445621; doi:10.2196/56804)
Supplement: Multimedia Appendix 1 [file jmir_v26i1e56804_app1.docx]

# **Supplemental File 1. List of Reviewed Maternal Health Dashboards**

| No. | Unique Name of Dashboard | Link to Dashboard^1^ |
| --- | --- | --- |
| *Dashboards with a national or multinational scope* (n = 8) | | |
| 1 | Agency for Healthcare Research and Quality: Perinatal Dashboard | [Direct Link](https://www.ahrq.gov/npsd/data/dashboard/perinatal.html) |
| 2 | Cesareanrates.org: State Dashboards | [Direct Link](https://www.cesareanrates.org/) |
| 3 | Health Resources and Services Administration: Maternal and Infant Health Mapping Tool | [Direct Link](https://data.hrsa.gov/maps/mchb/) |
| 4 | Kaiser Family Foundation: State Profiles for Women's Health | [Direct Link](https://www.kff.org/interactive/womens-health-profiles/?activeState=United%20States&activeCategory=abortion-policies) |
| 5 | March of Dimes: PeriStats^TM^ | [Direct Link](https://www.marchofdimes.org/peristats/) |
| 6 | Power to Decide: National Data | n/a |
| 7 | UNICEF: Child Health and Wellbeing Dashboard | [Direct Link](https://data.unicef.org/resources/child-health-and-well-being-dashboard/) |
| 8 | University of Wisconsin Population Health Institute: County Health Rankings | [Direct Link](https://www.countyhealthrankings.org/) |
| *Dashboards with a state-level scope* (n = 58) | | |
| 9 | Alabama: Alabama Health Dashboard | [Direct Link](https://www.alabamapublichealth.gov/healthrankings/dashboard.html) |
| 10 | Alabama: Alabama Perinatal Quality Collaborative Perinatal Resources Directory | [Direct Link](https://uabsoph.maps.arcgis.com/apps/MapJournal/index.html?appid=d6fca1b6b56f44f4be7924761893af45) |
| 11 | Alaska: Maternal and Child Health Indicators | [Direct Link](https://mch-indicators2-alaska-dhss.hub.arcgis.com/) |
| 12 | California: Department of Public Health Maternal, Child, and Adolescent Health Division Data Dashboards | [Direct Link](https://www.cdph.ca.gov/Programs/CFH/DMCAH/surveillance/Pages/default.aspx#backtoTop) |
| 13 | Colorado: Pregnancy Risk Assessment Monitoring System (PRAMS) Prevalence Estimates, 2021 | [Direct Link](https://cohealthviz.dphe.state.co.us/t/HSEBPublic/views/2021TableauSummaryTables/2021PRAMSSummaryTables?%3Aembed=y&%3AisGuestRedirectFromVizportal=y&%3Amobile=true) |
| 14 | Connecticut: Healthy Connecticut 2020 Performance Dashboard | [Direct Link](https://stateofhealth.ct.gov/HCT2020) |
| 15 | Delaware: My Healthy Community Environmental Public Health Tracking Network | [Direct Link](https://myhealthycommunity.dhss.delaware.gov/locations/state/maternal-and-child-health/) |
| 16 | Florida: Florida Health Community Health Assessment Resource Tool Set (FLHealthCHARTS) Maternal and Child Health | [Direct Link](https://www.flhealthcharts.gov/charts/MaternalAndChildHealth/default.aspx) |
| 17 | Georgia: Maternal Health 2008-2018 | [Direct Link](https://www.arcgis.com/apps/mapviewer/index.html?layers=670aaca52fb44b14b75f291df5083401) |
| 18 | Georgia: Online Analytical Statistical Information System (OASIS) - Maternal and Child Health | [Direct Link](https://oasis.state.ga.us/) |
| 19 | Hawaii: Health Data Warehouse/Hawaii Health Matters | [Direct Link](https://hhdw.org/) |
| 20 | Idaho: Vital Statistics Natality Dashboard | [Direct Link](https://www.gethealthy.dhw.idaho.gov/idaho-births-vital-statistics) |
| 21 | Illinois: PRAMS | [Direct Link](https://dph.illinois.gov/data-statistics/pregnancy-risk-assessment-monitoring-system/2020/table-1.html) |
| 22 | Illinois: University of Illinois Chicago Innovations to Improve Maternal Outcomes in Illinois (I PROMOTE-IL) Dashboard | [Direct Link](https://ipromoteil.org/) |
| 23 | Illinois: Vital Statistics | [Direct Link](https://dph.illinois.gov/data-statistics/vital-statistics.html) |
| 24 | Indiana: Department of Health Interactive Query Tools and Dashboards | n/a |
| 25 | Iowa: Public Health Tracking Portal | [Direct Link](https://tracking.idph.iowa.gov/People-Community/reproduction-and-birth/Reproductive-Outcomes-and-the-Environment) |
| 26 | Iowa: State Data Center Medicaid Reimbursed Births | [Direct Link](https://www.iowadatacenter.org/index.php) |
| 27 | Kansas: Kansas Health Matters | [Direct Link](https://www.kansashealthmatters.org/indicators) |
| 28 | Kentucky: Kids Count Data Dashboard | [Direct Link](https://kyyouth.org/kentucky-kids-count/data/) |
| 29 | Louisiana: Department of Health Medicaid Managed Care Quality Dashboard | [Direct Link](https://qualitydashboard.ldh.la.gov/) |
| 30 | Maine: State Epidemiologic Outcomes Workgroup, Substance Use Among Pregnant and Postpartum Individuals and Substance Exposed Infants Dashboard | n/a |
| 31 | Maine: State of Maine Data, Research, and Vital Statistics Maternal and Birth Data Dashboard | [Direct Link](https://www.maine.gov/dhhs/mecdc/public-health-systems/data-research/vital-records/births.shtml) |
| 32 | Maryland: Maryland Maternal Health Innovation Program (MDMOM) Data Dashboard | n/a |
| 33 | Massachusetts: Department of Public Health Maternal Analytics | [Direct Link](https://dphanalytics.hhs.mass.gov/ibmcognos/bi/?perspective=authoring&pathRef=.public_folders%2FMEPHTN%2Fdph%2FPHIT-Reports%2Fprams-80&id=i95598CC84098407B92E2783DB529D4DC&ui_appbar=false&ui_navbar=false&objRef=i95598CC84098407B92E2783DB529D4DC&action=run&format=HTML&cmPropStr=%7B%22id%22%3A%22i95598CC84098407B92E2783DB529D4DC%22%2C%22type%22%3A%22report%22%2C%22defaultName%22%3A%22prams-80%22%2C%22permissions%22%3A%5B%22execute%22%2C%22read%22%2C%22traverse%22%5D%7D) |
| 34 | Massachusetts: Neonatal Abstinence Syndrome Dashboard | [Direct Link](https://www.mass.gov/info-details/neonatal-abstinence-syndrome-data) |
| 35 | Michigan: Natality, Pregnancy, and Abortion Statistics | [Direct Link](https://vitalstats.michigan.gov/osr/index.asp?Id=2) |
| 36 | Minnesota: Public Health Data Access Portal Birth Defects Dashboard | [Direct Link](https://data.web.health.state.mn.us/birthoutcomes_query) |
| 37 | Missouri: HealthNet Managed Care Quality Dashboard | [Direct Link](https://dss.mo.gov/mhd/mc/pages/dashboard.htm) |
| 38 | Missouri: Pregnancy-Associated Mortality Review Dashboard 2017-19 | [Direct Link](https://health.mo.gov/data/pamr/dashboard.php) |
| 39 | Montana: Department of Public Health and Human Services Healthy Montana Families (HMF) Needs Assessment Dashboard | [Direct Link](https://dphhs.mt.gov/InteractiveDashboards/HMFNeedsAssessment) |
| 40 | Montana: Department of Public Health and Human Services Montana Medicaid Births Dashboard | [Direct Link](https://dphhs.mt.gov/interactivedashboards/mtmedicaidbirthsdashboard) |
| 41 | Montana: Department of Public Health and Human Services PRAMS Indicators Dashboard | [Direct Link](https://dphhs.mt.gov/InteractiveDashboards/PRAMSDashboard) |
| 42 | Montana: Kids Count Montana Children's Health Dashboard | [Direct Link](https://montanakidscount.org/montana-childrens-health-data-dashboard) |
| 43 | Nebraska: Healthy People 2020: Maternal, Infant and Child Health | [Direct Link](https://embed.clearimpact.com/Result/Embed/9597) |
| 44 | Nevada: Maternal and Perinatal Health Dashboard | [Direct Link](https://app.powerbigov.us/view?r=eyJrIjoiZTBmY2Y0OTgtOGFiOS00OTEyLWFkM2QtNmQ2NTI1YjI5YjgzIiwidCI6ImU0YTM0MGU2LWI4OWUtNGU2OC04ZWFhLTE1NDRkMjcwMzk4MCJ9) |
| 45 | New Hampshire: Health and Human Services Data Portal Pregnancy Dashboard | [Direct Link](https://wisdom.dhhs.nh.gov/wisdom/topics.html?topic=pregnancy) |
| 46 | New Jersey: Department of Health Maternal Health Hospital Report Card | [Direct Link](https://www.nj.gov/health/maternal/morbidity/mhh_reportcard/2018-2019/index.shtml) |
| 47 | New Mexico: Environmental and Public Health Tracking Data Portal | [Direct Link](https://nmtracking.doh.nm.gov/dataportal/indicator/Index.html) |
| 48 | New York State: Maternal and Child Health (MCH) Dashboard | [Direct Link](https://webbi1.health.ny.gov/SASStoredProcess/guest?_program=/EBI/PHIG/apps/mch_dashboard/mch_dashboard&p=sh) |
| 49 | North Carolina: NC Child's County Data Cards & Interactive Data Dashboard | [Direct Link](https://ncchild.org/what-we-do/insights/data/2021county-data-cards/) |
| 50 | Ohio: The Ohio Pregnancy Assessment Survey (OPAS) Series Dashboard | [Direct Link](https://grcapps.osu.edu/app/opas) |
| 51 | Oregon: Oregon Health Authority Vital Statistics Annual Report Birth & Pregnancy Dashboards | [Direct Link](https://www.oregon.gov/oha/PH/BIRTHDEATHCERTIFICATES/VITALSTATISTICS/ANNUALREPORTS/Pages/index.aspx) |
| 52 | Rhode Island: Department of Health Maternal and Child Health Data | [Direct Link](https://health.ri.gov/data/maternalandchildhealth/) |
| 53 | Rhode Island: Department of Health Pregnancy Risk Assessment Monitoring System Data | [Direct Link](https://health.ri.gov/data/pregnancyriskassessment/) |
| 54 | South Carolina: University of South Carolina Institute for Families in Society Birth Outcomes Initiative | [Direct Link](https://boi.ifsreports.com/statewide/maternalhealth.html) |
| 55 | Tennessee: Department of Health Pregnancy Risk Assessment Monitoring System Dashboard | [Direct Link](https://www.tn.gov/health/population-health--assessment/prams/dashboard.html) |
| 56 | Texas: Texas Health Data Pregnancy Risk Assessment Monitoring System | [Direct Link](https://healthdata.dshs.texas.gov/dashboard/surveys-and-profiles/pregnancy-risk-assessment-monitoring-system) |
| 57 | Utah: Public Health Indicator-Based Information System Births and Maternity | [Direct Link](https://ibis.health.utah.gov/ibisph-view/topic/Births.html) |
| 58 | Utah: Public Health Indicator-Based Information System PRAMS | [Direct Link](https://ibis.health.utah.gov/ibisph-view/query/selection/prams/PRAMSSelection.html) |
| 59 | Vermont: Maternal and Infant Health Performance Dashboard | [Direct Link](https://embed.clearimpact.com/Scorecard/Embed/604) |
| 60 | Virginia: Department of Health Maternal and Child Health Dashboard | n/a |
| 61 | Virginia: Department of Health PRAMS Data Dashboard | [Direct Link](https://www.vdh.virginia.gov/prams/data-dashboards/) |
| 62 | Washington: Department of Health Washington Tracking Network (WTN) Birth Outcomes Data Dashboards | [Direct Link](https://doh.wa.gov/data-and-statistical-reports/washington-tracking-network-wtn/birth-outcomes-data) |
| 63 | Washington: Department of Health Washington Tracking Network (WTN) Environmental Public Health Data | [Direct Link](https://fortress.wa.gov/doh/wtn/WTNPortal/home/) |
| 64 | Washington: Washington State Health Care Authority Medicaid Maternal and Child Health Measures Dashboard | [Direct Link](https://hca-tableau.watech.wa.gov/t/51/views/MaternalandChildHealth/Dashboard?%3AisGuestRedirectFromVizportal=y&%3Aembed=y) |
| 65 | Wisconsin: Wisconsin Interactive Statistics on Health (WISH) | [Direct Link](https://dhs.wisconsin.gov/wish/index.htm) |
| 66 | Wyoming: Maternal and Child Health Vital Statistics Services Trend Reports | [Direct Link](https://sites.google.com/wyo.gov/wymchvsstrendreports/home) |
| 67 | Wyoming: Wyoming MCH Title V National Outcome Measures (NOM) and National Performance Measures (NPM) | [Direct Link](https://sites.google.com/wyo.gov/title-v-national-om-pm/home) |
| *Dashboards with a regional scope^2^* (n = 13) | | |
| 68 | Central Oregon Health Data | [Direct Link](https://www.centraloregonhealthdata.org/indicators/index/dashboard?alias=indicatorlist) |
| 69 | Great Plains Area Maternal and Child Health by Race, 2014-2018 (Tribal Areas) | [Direct Link](https://public.tableau.com/app/profile/sarah.shewbrooks/viz/MCH22014to2018/MCH2014-2018?publish=yes) |
| 70 | Health Compass Milwaukee (Wisconsin) | [Direct Link](https://www.healthcompassmilwaukee.org/index.php?controller=index&module=indicators&action=indicatorsearch&doSearch=1&handpicked=1&i=172_173_289_325_328_6051_8619_292_430_11111) |
| 71 | Healthy Northeast Ohio | [Direct Link](https://www.healthyneo.org/indicators/index/indicatorsearch?module=indicators&controller=index&action=indicatorsearch&doSearch=1&i=&l=&t%5B%5D=61&primaryTopicOnly=&subgrouping=3&card=0&handpicked=0&resultsPerPage=150&showComparisons=1&showOnlySelectedComparisons=&showOnlySelectedComparisons=1&includeArchivedIndicators=&includeArchivedIndicators=1&grouping=1&ordering=1&sortcomp=0&sortcompIncludeMissing=) |
| 72 | Healthy Southern Nevada Community Dashboard | [Direct Link](https://www.healthysouthernnevada.org/indicators/index/indicatorsearch?module=indicators&controller=index&action=indicatorsearch&doSearch=1&i=&l=&t%5B%5D=61&primaryTopicOnly=&subgrouping=3&card=0&handpicked=0&resultsPerPage=150&showComparisons=1&showOnlySelectedComparisons=&showOnlySelectedComparisons=1&includeArchivedIndicators=&includeArchivedIndicators=1&grouping=1&ordering=1&sortcomp=0&sortcompIncludeMissing=) |
| 73 | Healthy Williamson County (Texas) | [Direct Link](https://www.healthywilliamsoncounty.org/index.php?module=indicators&controller=index&action=dashboard&alias=maternalhealth) |
| 74 | Maricopa County Community Health Assessment (Arizona) | [Direct Link](https://dashboards.mysidewalk.com/maricopa-county-az-community-health-assessment/infant-and-maternal-health) |
| 75 | Michigan Health Improvement Alliance (Great Lakes Bay Region) | [Direct Link](https://dashboard.mihia.org/indicators/index/view?indicatorId=430&localeId=1338) |
| 76 | New Mexico State University Center for Community Analysis Maternal, Infant, and Child Health Data by New Mexico County (New Mexico) | [Direct Link](https://cca.nmsu.edu/dashboards-list/maternal-health.html) |
| 77 | New York City: Health Data—Birth Trends | [Direct Link](https://a816-health.nyc.gov/hdi/epiquery/visualizations?PageType=ts&PopulationSource=Birth&Topic=8&Subtopic=38) |
| 78 | Pittsburgh Maternal Well-Being City Dashboard | [Direct Link](https://dashboards.mysidewalk.com/maternal-wellbeing-pittsburgh-pa/neighborhood-outcome) |
| 79 | Santa Fe Maternal and Child Health Dashboard (New Mexico) | [Direct Link](https://dashboards.mysidewalk.com/santa-fe-public-health-dashboard/welcome) |
| 80 | Summit County (Ohio) Public Health: Birth Certificate Dashboard | [Direct Link](https://app.powerbi.com/view?r=eyJrIjoiNDlmOGIzMWItZDMwZi00N2M5LWI3YjktZTQ2NDIyMTllMWEyIiwidCI6ImJiMWI0YjU2LTQ4N2EtNGIyMy04YTI0LWEzYWVmNjVlMTFmZiIsImMiOjF9) |

^1^Provided links were active as of May 2024; dashboards that became unavailable following the initial abstraction period (Feb-May 20203) are marked as n/a; ^2^Regional scope is defined as a geographic area smaller than a state or multiple geographic areas that span states (e.g., tribal areas).
