# Supplementary material for: Public Maternal Health Dashboards in the United States: Descriptive Assessment
Source: J Med Internet Res. 2024 Sep 17;26:e56804. doi: 10.2196/56804 (PMC11445621; doi:10.2196/56804)
Supplement: Multimedia Appendix 2 [file jmir_v26i1e56804_app2.docx]

**Supplemental File 2. REDCap Data Abstraction Instrument**

|  | **#** | **Variable/Field Name** | **Field Label**  ***Field Note*** | **Field Attributes (Field Type, Validation, Choices, Calculations, etc.)** |
| --- | --- | --- | --- | --- |
| [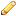](https://base.uams.edu/redcap/redcap_v13.1.29/Design/online_designer.php?pid=5429&page=dashboard_features_and_indicators&field=record_id) | 1 | [record_id] | Record ID | text |
| [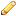](https://base.uams.edu/redcap/redcap_v13.1.29/Design/online_designer.php?pid=5429&page=dashboard_features_and_indicators&field=dashboard_uniqname)  [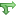](https://base.uams.edu/redcap/redcap_v13.1.29/Design/online_designer.php?pid=5429&page=dashboard_features_and_indicators&field=dashboard_uniqname&branching=1) | 2 | [dashboard_uniqname] | Section Header: *Section 1: Design and Features of Reviewed Maternal Health Dashboards*  Unique name of the dashboard | text, Required, Identifier Custom alignment: LV Question number: 1 |
| [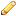](https://base.uams.edu/redcap/redcap_v13.1.29/Design/online_designer.php?pid=5429&page=dashboard_features_and_indicators&field=dashboard_url)  [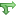](https://base.uams.edu/redcap/redcap_v13.1.29/Design/online_designer.php?pid=5429&page=dashboard_features_and_indicators&field=dashboard_url&branching=1) | 3 | [dashboard_url] | Dashboard URL | text, Required, Identifier Custom alignment: LV Question number: 2 |
| [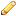](https://base.uams.edu/redcap/redcap_v13.1.29/Design/online_designer.php?pid=5429&page=dashboard_features_and_indicators&field=datascope)  [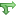](https://base.uams.edu/redcap/redcap_v13.1.29/Design/online_designer.php?pid=5429&page=dashboard_features_and_indicators&field=datascope&branching=1) | 4 | [datascope] | Scope of data included in dashboard | checkbox, Required   \| 1 \| datascope___1 \| Maternal/perinatal data only \| \| --- \| --- \| --- \| \| 2 \| datascope___2 \| Maternal/perinatal and child data \| \| 3 \| datascope___3 \| Broader than maternal and child (specify): {datascope_other} \|   Custom alignment: LV Question number: 3 |
| [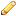](https://base.uams.edu/redcap/redcap_v13.1.29/Design/online_designer.php?pid=5429&page=dashboard_features_and_indicators&field=datascope_other)  [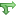](https://base.uams.edu/redcap/redcap_v13.1.29/Design/online_designer.php?pid=5429&page=dashboard_features_and_indicators&field=datascope_other&branching=1) | 5 | [datascope_other]  Show the field ONLY if:  [datascope(3)] = '1' | other scope of data | text, Required Custom alignment: LV Question number: 4 |
| [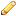](https://base.uams.edu/redcap/redcap_v13.1.29/Design/online_designer.php?pid=5429&page=dashboard_features_and_indicators&field=geoscope)  [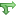](https://base.uams.edu/redcap/redcap_v13.1.29/Design/online_designer.php?pid=5429&page=dashboard_features_and_indicators&field=geoscope&branching=1) | 6 | [geoscope] | What is the PRIMARY geographic scope of the dashboard? | radio, Required   \| 1 \| National or Multinational \| \| --- \| --- \| \| 2 \| State \| \| 3 \| Region or other area (specify): {geoscope_other} \|   Custom alignment: LV Question number: 5 |
| [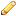](https://base.uams.edu/redcap/redcap_v13.1.29/Design/online_designer.php?pid=5429&page=dashboard_features_and_indicators&field=geoscope_other)  [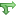](https://base.uams.edu/redcap/redcap_v13.1.29/Design/online_designer.php?pid=5429&page=dashboard_features_and_indicators&field=geoscope_other&branching=1) | 7 | [geoscope_other]  Show the field ONLY if:  [geoscope] = '3' | other geographical scope of data | text, Required Custom alignment: LV Question number: 6 |
| [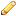](https://base.uams.edu/redcap/redcap_v13.1.29/Design/online_designer.php?pid=5429&page=dashboard_features_and_indicators&field=hosting_site)  [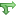](https://base.uams.edu/redcap/redcap_v13.1.29/Design/online_designer.php?pid=5429&page=dashboard_features_and_indicators&field=hosting_site&branching=1) | 8 | [hosting_site] | Hosting site of dashboard | radio, Required   \| 1 \| State health department \| \| --- \| --- \| \| 2 \| Nonprofit/program website \| \| 3 \| Other (specify) {hosting_site_other} \|   Custom alignment: LV Question number: 9 |
| [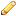](https://base.uams.edu/redcap/redcap_v13.1.29/Design/online_designer.php?pid=5429&page=dashboard_features_and_indicators&field=purpose)  [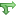](https://base.uams.edu/redcap/redcap_v13.1.29/Design/online_designer.php?pid=5429&page=dashboard_features_and_indicators&field=purpose&branching=1) | 9 | [purpose] | Statement defining PURPOSE of dashboard (does not have to be worded like an official statement) | yesno, Required   \| 1 \| Yes \| \| --- \| --- \| \| 0 \| No \|   Custom alignment: LV Question number: 7 |
| [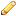](https://base.uams.edu/redcap/redcap_v13.1.29/Design/online_designer.php?pid=5429&page=dashboard_features_and_indicators&field=purposeexp)  [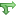](https://base.uams.edu/redcap/redcap_v13.1.29/Design/online_designer.php?pid=5429&page=dashboard_features_and_indicators&field=purposeexp&branching=1) | 10 | [purposeexp]  Show the field ONLY if:  [purpose] = '1' | Purpose Explanation (you can copy and paste short phrases and sentences from the dashboard) | notes |
| [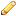](https://base.uams.edu/redcap/redcap_v13.1.29/Design/online_designer.php?pid=5429&page=dashboard_features_and_indicators&field=purposeurls)  [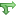](https://base.uams.edu/redcap/redcap_v13.1.29/Design/online_designer.php?pid=5429&page=dashboard_features_and_indicators&field=purposeurls&branching=1) | 11 | [purposeurls]  Show the field ONLY if:  [purpose] = '1' | Enter the URLs Related to Purpose | notes |
| [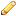](https://base.uams.edu/redcap/redcap_v13.1.29/Design/online_designer.php?pid=5429&page=dashboard_features_and_indicators&field=purpose_category)  [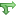](https://base.uams.edu/redcap/redcap_v13.1.29/Design/online_designer.php?pid=5429&page=dashboard_features_and_indicators&field=purpose_category&branching=1) | 12 | [purpose_category]  Show the field ONLY if:  [purpose]=1 | Dashboard purpose category | checkbox   \| 1 \| purpose_category___1 \| Data accessibility \| \| --- \| --- \| --- \| \| 2 \| purpose_category___2 \| Accountability for government/organizations \| \| 3 \| purpose_category___3 \| Individual Decision Making \| \| 4 \| purpose_category___4 \| Program Planning/ needs assessment \| \| 5 \| purpose_category___5 \| Program monitoring/ evaluation \| \| 6 \| purpose_category___6 \| Research \| \| 7 \| purpose_category___7 \| Policy making \| \| 8 \| purpose_category___8 \| Quality Improvement \| \| 99 \| purpose_category___99 \| Other \|   Custom alignment: LV |
| [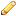](https://base.uams.edu/redcap/redcap_v13.1.29/Design/online_designer.php?pid=5429&page=dashboard_features_and_indicators&field=audience)  [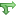](https://base.uams.edu/redcap/redcap_v13.1.29/Design/online_designer.php?pid=5429&page=dashboard_features_and_indicators&field=audience&branching=1) | 13 | [audience] | Statement explaining/defining AUDIENCE of dashboard? (does not have to be worded like an official statement) | yesno, Required   \| 1 \| Yes \| \| --- \| --- \| \| 0 \| No \|   Custom alignment: LV Question number: 8 |
| [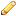](https://base.uams.edu/redcap/redcap_v13.1.29/Design/online_designer.php?pid=5429&page=dashboard_features_and_indicators&field=audienceexp)  [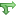](https://base.uams.edu/redcap/redcap_v13.1.29/Design/online_designer.php?pid=5429&page=dashboard_features_and_indicators&field=audienceexp&branching=1) | 14 | [audienceexp]  Show the field ONLY if:  [audience] = '1' | Audience Explanation (you can copy-paste short phrases or sentences from the dashboard website) | notes |
| [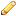](https://base.uams.edu/redcap/redcap_v13.1.29/Design/online_designer.php?pid=5429&page=dashboard_features_and_indicators&field=audienceurls)  [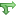](https://base.uams.edu/redcap/redcap_v13.1.29/Design/online_designer.php?pid=5429&page=dashboard_features_and_indicators&field=audienceurls&branching=1) | 15 | [audienceurls]  Show the field ONLY if:  [audience] = '1' | Enter the URLs Related to Audience | notes |
| [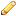](https://base.uams.edu/redcap/redcap_v13.1.29/Design/online_designer.php?pid=5429&page=dashboard_features_and_indicators&field=audience_category)  [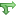](https://base.uams.edu/redcap/redcap_v13.1.29/Design/online_designer.php?pid=5429&page=dashboard_features_and_indicators&field=audience_category&branching=1) | 16 | [audience_category]  Show the field ONLY if:  [audience]=1 | Dashboard audience category | checkbox   \| 1 \| audience_category___1 \| General audience descriptor \| \| --- \| --- \| --- \| \| 2 \| audience_category___2 \| Healthcare organizations/providers \| \| 3 \| audience_category___3 \| Public health agencies/organizations \| \| 4 \| audience_category___4 \| Legislators/policy makers \| \| 5 \| audience_category___5 \| Community members/healthcare consumers \| \| 6 \| audience_category___6 \| Researchers \| \| 7 \| audience_category___7 \| Other \|   Custom alignment: LV |
| [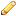](https://base.uams.edu/redcap/redcap_v13.1.29/Design/online_designer.php?pid=5429&page=dashboard_features_and_indicators&field=hosting_site_other)  [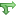](https://base.uams.edu/redcap/redcap_v13.1.29/Design/online_designer.php?pid=5429&page=dashboard_features_and_indicators&field=hosting_site_other&branching=1) | 17 | [hosting_site_other]  Show the field ONLY if:  [hosting_site] = '3' | Please specify if other hosting site. | text, Required Question number: 10 |
| [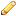](https://base.uams.edu/redcap/redcap_v13.1.29/Design/online_designer.php?pid=5429&page=dashboard_features_and_indicators&field=disagg_geo&matrix=1)  [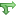](https://base.uams.edu/redcap/redcap_v13.1.29/Design/online_designer.php?pid=5429&page=dashboard_features_and_indicators&field=disagg_geo&branching=1) | 18 | [disagg_geo] | Section Header: *Section 1: Design and Features of Reviewed Maternal Health Dashboards Disaggregation capabilities...*  ...by geography | radio (Matrix), Required   \| 1 \| No \| \| --- \| --- \| \| 2 \| Yes, for some data \| \| 3 \| Yes, for all data \| |
| [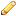](https://base.uams.edu/redcap/redcap_v13.1.29/Design/online_designer.php?pid=5429&page=dashboard_features_and_indicators&field=disagg_race&matrix=1)  [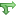](https://base.uams.edu/redcap/redcap_v13.1.29/Design/online_designer.php?pid=5429&page=dashboard_features_and_indicators&field=disagg_race&branching=1) | 19 | [disagg_race] | ...by race/ethnicity | radio (Matrix), Required   \| 1 \| No \| \| --- \| --- \| \| 2 \| Yes, for some data \| \| 3 \| Yes, for all data \| |
| [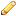](https://base.uams.edu/redcap/redcap_v13.1.29/Design/online_designer.php?pid=5429&page=dashboard_features_and_indicators&field=disagg_mothers_age&matrix=1)  [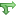](https://base.uams.edu/redcap/redcap_v13.1.29/Design/online_designer.php?pid=5429&page=dashboard_features_and_indicators&field=disagg_mothers_age&branching=1) | 20 | [disagg_mothers_age] | ...by mother's age or age group | radio (Matrix), Required   \| 1 \| No \| \| --- \| --- \| \| 2 \| Yes, for some data \| \| 3 \| Yes, for all data \| |
| [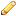](https://base.uams.edu/redcap/redcap_v13.1.29/Design/online_designer.php?pid=5429&page=dashboard_features_and_indicators&field=disagg_education&matrix=1)  [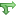](https://base.uams.edu/redcap/redcap_v13.1.29/Design/online_designer.php?pid=5429&page=dashboard_features_and_indicators&field=disagg_education&branching=1) | 21 | [disagg_education] | ...by education | radio (Matrix), Required   \| 1 \| No \| \| --- \| --- \| \| 2 \| Yes, for some data \| \| 3 \| Yes, for all data \| |
| [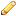](https://base.uams.edu/redcap/redcap_v13.1.29/Design/online_designer.php?pid=5429&page=dashboard_features_and_indicators&field=disagg_marital_status&matrix=1)  [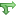](https://base.uams.edu/redcap/redcap_v13.1.29/Design/online_designer.php?pid=5429&page=dashboard_features_and_indicators&field=disagg_marital_status&branching=1) | 22 | [disagg_marital_status] | ...by marital status | radio (Matrix), Required   \| 1 \| No \| \| --- \| --- \| \| 2 \| Yes, for some data \| \| 3 \| Yes, for all data \| |
| [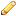](https://base.uams.edu/redcap/redcap_v13.1.29/Design/online_designer.php?pid=5429&page=dashboard_features_and_indicators&field=disagg_sex&matrix=1)  [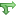](https://base.uams.edu/redcap/redcap_v13.1.29/Design/online_designer.php?pid=5429&page=dashboard_features_and_indicators&field=disagg_sex&branching=1) | 23 | [disagg_sex] | ...by sex or gender of mother/baby | radio (Matrix), Required   \| 1 \| No \| \| --- \| --- \| \| 2 \| Yes, for some data \| \| 3 \| Yes, for all data \| |
| [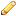](https://base.uams.edu/redcap/redcap_v13.1.29/Design/online_designer.php?pid=5429&page=dashboard_features_and_indicators&field=disagg_plurality&matrix=1)  [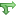](https://base.uams.edu/redcap/redcap_v13.1.29/Design/online_designer.php?pid=5429&page=dashboard_features_and_indicators&field=disagg_plurality&branching=1) | 24 | [disagg_plurality] | ...by plurality | radio (Matrix), Required   \| 1 \| No \| \| --- \| --- \| \| 2 \| Yes, for some data \| \| 3 \| Yes, for all data \| |
| [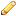](https://base.uams.edu/redcap/redcap_v13.1.29/Design/online_designer.php?pid=5429&page=dashboard_features_and_indicators&field=disagg_bw&matrix=1)  [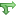](https://base.uams.edu/redcap/redcap_v13.1.29/Design/online_designer.php?pid=5429&page=dashboard_features_and_indicators&field=disagg_bw&branching=1) | 25 | [disagg_bw] | ...by birthweight | radio (Matrix), Required   \| 1 \| No \| \| --- \| --- \| \| 2 \| Yes, for some data \| \| 3 \| Yes, for all data \| |
| [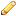](https://base.uams.edu/redcap/redcap_v13.1.29/Design/online_designer.php?pid=5429&page=dashboard_features_and_indicators&field=disagg_g_age&matrix=1)  [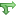](https://base.uams.edu/redcap/redcap_v13.1.29/Design/online_designer.php?pid=5429&page=dashboard_features_and_indicators&field=disagg_g_age&branching=1) | 26 | [disagg_g_age] | ...by gestational age | radio (Matrix), Required   \| 1 \| No \| \| --- \| --- \| \| 2 \| Yes, for some data \| \| 3 \| Yes, for all data \| |
| [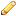](https://base.uams.edu/redcap/redcap_v13.1.29/Design/online_designer.php?pid=5429&page=dashboard_features_and_indicators&field=disagg_income&matrix=1)  [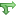](https://base.uams.edu/redcap/redcap_v13.1.29/Design/online_designer.php?pid=5429&page=dashboard_features_and_indicators&field=disagg_income&branching=1) | 27 | [disagg_income] | ...by household income | radio (Matrix), Required   \| 1 \| No \| \| --- \| --- \| \| 2 \| Yes, for some data \| \| 3 \| Yes, for all data \| |
| [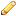](https://base.uams.edu/redcap/redcap_v13.1.29/Design/online_designer.php?pid=5429&page=dashboard_features_and_indicators&field=disagg_poverty&matrix=1)  [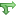](https://base.uams.edu/redcap/redcap_v13.1.29/Design/online_designer.php?pid=5429&page=dashboard_features_and_indicators&field=disagg_poverty&branching=1) | 28 | [disagg_poverty] | ...by level of poverty | radio (Matrix), Required   \| 1 \| No \| \| --- \| --- \| \| 2 \| Yes, for some data \| \| 3 \| Yes, for all data \| |
| [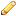](https://base.uams.edu/redcap/redcap_v13.1.29/Design/online_designer.php?pid=5429&page=dashboard_features_and_indicators&field=disagg_by_time&matrix=1)  [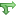](https://base.uams.edu/redcap/redcap_v13.1.29/Design/online_designer.php?pid=5429&page=dashboard_features_and_indicators&field=disagg_by_time&branching=1) | 29 | [disagg_by_time] | ... by time interval (e.g. year) | radio (Matrix), Required   \| 1 \| No \| \| --- \| --- \| \| 2 \| Yes, for some data \| \| 3 \| Yes, for all data \| |
| [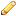](https://base.uams.edu/redcap/redcap_v13.1.29/Design/online_designer.php?pid=5429&page=dashboard_features_and_indicators&field=disagg_insurance&matrix=1)  [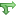](https://base.uams.edu/redcap/redcap_v13.1.29/Design/online_designer.php?pid=5429&page=dashboard_features_and_indicators&field=disagg_insurance&branching=1) | 30 | [disagg_insurance] | ...by insurance type/status | radio (Matrix), Required   \| 1 \| No \| \| --- \| --- \| \| 2 \| Yes, for some data \| \| 3 \| Yes, for all data \| |
| [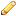](https://base.uams.edu/redcap/redcap_v13.1.29/Design/online_designer.php?pid=5429&page=dashboard_features_and_indicators&field=disagg_facility&matrix=1)  [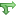](https://base.uams.edu/redcap/redcap_v13.1.29/Design/online_designer.php?pid=5429&page=dashboard_features_and_indicators&field=disagg_facility&branching=1) | 31 | [disagg_facility] | ...by healthcare facility | radio (Matrix), Required   \| 1 \| No \| \| --- \| --- \| \| 2 \| Yes, for some data \| \| 3 \| Yes, for all data \| |
| [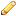](https://base.uams.edu/redcap/redcap_v13.1.29/Design/online_designer.php?pid=5429&page=dashboard_features_and_indicators&field=disagg_provider&matrix=1)  [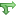](https://base.uams.edu/redcap/redcap_v13.1.29/Design/online_designer.php?pid=5429&page=dashboard_features_and_indicators&field=disagg_provider&branching=1) | 32 | [disagg_provider] | ...by healthcare provider | radio (Matrix), Required   \| 1 \| No \| \| --- \| --- \| \| 2 \| Yes, for some data \| \| 3 \| Yes, for all data \| |
| [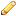](https://base.uams.edu/redcap/redcap_v13.1.29/Design/online_designer.php?pid=5429&page=dashboard_features_and_indicators&field=disagg_social_support&matrix=1)  [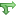](https://base.uams.edu/redcap/redcap_v13.1.29/Design/online_designer.php?pid=5429&page=dashboard_features_and_indicators&field=disagg_social_support&branching=1) | 33 | [disagg_social_support] | ...by receipt of social support (WIC, SNAP, etc.) {social_support} | radio (Matrix), Required   \| 1 \| No \| \| --- \| --- \| \| 2 \| Yes, for some data \| \| 3 \| Yes, for all data \| |
| [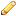](https://base.uams.edu/redcap/redcap_v13.1.29/Design/online_designer.php?pid=5429&page=dashboard_features_and_indicators&field=disagg_nativity&matrix=1)  [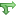](https://base.uams.edu/redcap/redcap_v13.1.29/Design/online_designer.php?pid=5429&page=dashboard_features_and_indicators&field=disagg_nativity&branching=1) | 34 | [disagg_nativity] | ...by nativity | radio (Matrix), Required   \| 1 \| No \| \| --- \| --- \| \| 2 \| Yes, for some data \| \| 3 \| Yes, for all data \| |
| [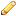](https://base.uams.edu/redcap/redcap_v13.1.29/Design/online_designer.php?pid=5429&page=dashboard_features_and_indicators&field=disagg_other_char&matrix=1)  [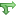](https://base.uams.edu/redcap/redcap_v13.1.29/Design/online_designer.php?pid=5429&page=dashboard_features_and_indicators&field=disagg_other_char&branching=1) | 35 | [disagg_other_char] | ...by other characteristics (specify) {disagg_other} | radio (Matrix), Required   \| 1 \| No \| \| --- \| --- \| \| 2 \| Yes, for some data \| \| 3 \| Yes, for all data \| |
| [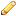](https://base.uams.edu/redcap/redcap_v13.1.29/Design/online_designer.php?pid=5429&page=dashboard_features_and_indicators&field=disagg_other)  [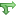](https://base.uams.edu/redcap/redcap_v13.1.29/Design/online_designer.php?pid=5429&page=dashboard_features_and_indicators&field=disagg_other&branching=1) | 36 | [disagg_other]  Show the field ONLY if:  [disagg_other_char] = '2' or [disagg_other_char] = '3' | Other disaggregation capabilities | text, Required Question number: 12 |
| [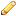](https://base.uams.edu/redcap/redcap_v13.1.29/Design/online_designer.php?pid=5429&page=dashboard_features_and_indicators&field=social_support)  [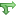](https://base.uams.edu/redcap/redcap_v13.1.29/Design/online_designer.php?pid=5429&page=dashboard_features_and_indicators&field=social_support&branching=1) | 37 | [social_support]  Show the field ONLY if:  [disagg_social_support] = '2' or [disagg_social_support] = '3' |  | text |
| [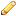](https://base.uams.edu/redcap/redcap_v13.1.29/Design/online_designer.php?pid=5429&page=dashboard_features_and_indicators&field=data_source)  [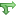](https://base.uams.edu/redcap/redcap_v13.1.29/Design/online_designer.php?pid=5429&page=dashboard_features_and_indicators&field=data_source&branching=1) | 38 | [data_source] | Reported Data Sources (select all that apply) | checkbox, Required   \| 1 \| data_source___1 \| Maternal Mortality Reviews \| \| --- \| --- \| --- \| \| 2 \| data_source___2 \| Vital Statistics \| \| 3 \| data_source___3 \| Hospital Discharge Data \| \| 4 \| data_source___4 \| Claims Data \| \| 5 \| data_source___5 \| PRAMS \| \| 7 \| data_source___7 \| HRSA \| \| 8 \| data_source___8 \| State Health Departments \| \| 9 \| data_source___9 \| US Census \| \| 10 \| data_source___10 \| National Center for Health Statistics \| \| 11 \| data_source___11 \| CDC Wonder \| \| 6 \| data_source___6 \| Other (specify) {other_data_source} \|   Custom alignment: LV Question number: 13 |
| [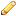](https://base.uams.edu/redcap/redcap_v13.1.29/Design/online_designer.php?pid=5429&page=dashboard_features_and_indicators&field=other_data_source)  [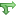](https://base.uams.edu/redcap/redcap_v13.1.29/Design/online_designer.php?pid=5429&page=dashboard_features_and_indicators&field=other_data_source&branching=1) | 39 | [other_data_source]  Show the field ONLY if:  [data_source(6)] = '1' | Other data sources | text, Required Custom alignment: LV Question number: 14 |
| [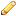](https://base.uams.edu/redcap/redcap_v13.1.29/Design/online_designer.php?pid=5429&page=dashboard_features_and_indicators&field=viz_types)  [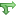](https://base.uams.edu/redcap/redcap_v13.1.29/Design/online_designer.php?pid=5429&page=dashboard_features_and_indicators&field=viz_types&branching=1) | 40 | [viz_types] | Types of visualizations (select all that apply) | checkbox, Required   \| 1 \| viz_types___1 \| Table \| \| --- \| --- \| --- \| \| 2 \| viz_types___2 \| Line graph \| \| 3 \| viz_types___3 \| Bar chart \| \| 4 \| viz_types___4 \| Map \| \| 6 \| viz_types___6 \| Pie chart \| \| 7 \| viz_types___7 \| Gauge chart \| \| 8 \| viz_types___8 \| Visual comparisons/change over time \| \| 9 \| viz_types___9 \| Large icons \| \| 5 \| viz_types___5 \| Other visualizations (specify) {other_viz_types} \|   Custom alignment: LV Question number: 15 |
| [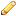](https://base.uams.edu/redcap/redcap_v13.1.29/Design/online_designer.php?pid=5429&page=dashboard_features_and_indicators&field=other_viz_types)  [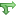](https://base.uams.edu/redcap/redcap_v13.1.29/Design/online_designer.php?pid=5429&page=dashboard_features_and_indicators&field=other_viz_types&branching=1) | 41 | [other_viz_types]  Show the field ONLY if:  [viz_types(5)] = '1' | Other visualizations used | text, Required Custom alignment: LV Question number: 16 |
| [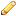](https://base.uams.edu/redcap/redcap_v13.1.29/Design/online_designer.php?pid=5429&page=dashboard_features_and_indicators&field=comparisons)  [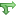](https://base.uams.edu/redcap/redcap_v13.1.29/Design/online_designer.php?pid=5429&page=dashboard_features_and_indicators&field=comparisons&branching=1) | 42 | [comparisons] | Available comparisons (select all that apply) | checkbox, Required   \| 1 \| comparisons___1 \| Other states/geographies (specify) {other_geo_comp} \| \| --- \| --- \| --- \| \| 2 \| comparisons___2 \| National data \| \| 3 \| comparisons___3 \| Benchmarks \| \| 4 \| comparisons___4 \| Longitudinal (e.g., previous years) \| \| 98 \| comparisons___98 \| None \|   Custom alignment: LV Question number: 17 Field Annotation: @NONEOFTHEABOVE=98 |
| [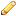](https://base.uams.edu/redcap/redcap_v13.1.29/Design/online_designer.php?pid=5429&page=dashboard_features_and_indicators&field=tech_capabilities)  [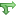](https://base.uams.edu/redcap/redcap_v13.1.29/Design/online_designer.php?pid=5429&page=dashboard_features_and_indicators&field=tech_capabilities&branching=1) | 43 | [tech_capabilities] | Technical capabilities in the dashboard | checkbox, Required   \| 1 \| tech_capabilities___1 \| All indicators visible on one page \| \| --- \| --- \| --- \| \| 2 \| tech_capabilities___2 \| User can download dashboard view (e.g., PDF) \| \| 3 \| tech_capabilities___3 \| User can download data \| \| 4 \| tech_capabilities___4 \| User can select visualization type \| \| 5 \| tech_capabilities___5 \| User can download visualizations \| \| 6 \| tech_capabilities___6 \| Responsive interface \| \| 7 \| tech_capabilities___7 \| Mobile-friendly interface \| \| 8 \| tech_capabilities___8 \| Full screen \| \| 9 \| tech_capabilities___9 \| Adaptive visualization (select to filter, etc.) \| \| 99 \| tech_capabilities___99 \| Other capabilities (specify) {other_tech_capabilities} \| \| 98 \| tech_capabilities___98 \| No specific technical capabilities \|   Custom alignment: LV Question number: 19 Field Annotation: @NONEOFTHEABOVE=98 |
| [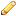](https://base.uams.edu/redcap/redcap_v13.1.29/Design/online_designer.php?pid=5429&page=dashboard_features_and_indicators&field=other_geo_comp)  [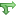](https://base.uams.edu/redcap/redcap_v13.1.29/Design/online_designer.php?pid=5429&page=dashboard_features_and_indicators&field=other_geo_comp&branching=1) | 44 | [other_geo_comp]  Show the field ONLY if:  [comparisons(1)] = '1' | other geographic comparisons | text, Required Custom alignment: LV Question number: 18 |
| [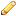](https://base.uams.edu/redcap/redcap_v13.1.29/Design/online_designer.php?pid=5429&page=dashboard_features_and_indicators&field=other_tech_capabilities)  [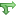](https://base.uams.edu/redcap/redcap_v13.1.29/Design/online_designer.php?pid=5429&page=dashboard_features_and_indicators&field=other_tech_capabilities&branching=1) | 45 | [other_tech_capabilities]  Show the field ONLY if:  [tech_capabilities(99)] = '1' | other tech capabilities | text, Required Question number: 20 |
| [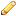](https://base.uams.edu/redcap/redcap_v13.1.29/Design/online_designer.php?pid=5429&page=dashboard_features_and_indicators&field=dash_software)  [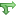](https://base.uams.edu/redcap/redcap_v13.1.29/Design/online_designer.php?pid=5429&page=dashboard_features_and_indicators&field=dash_software&branching=1) | 46 | [dash_software] | Dashboard's software platform | radio, Required   \| 1 \| Tableau \| \| --- \| --- \| \| 2 \| PowerBI \| \| 4 \| ArcGIS \| \| 5 \| Proprietary [Conduent technologies] \| \| 6 \| Proprietary [Clear Impact] \| \| 7 \| Proprietary [MySidewalk] \| \| 8 \| Proprietary [IBM Cognos] \| \| 3 \| Other, specify {other_software} \| \| 99 \| Not specified \|   Custom alignment: LV Question number: 21 |
| [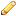](https://base.uams.edu/redcap/redcap_v13.1.29/Design/online_designer.php?pid=5429&page=dashboard_features_and_indicators&field=other_software)  [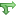](https://base.uams.edu/redcap/redcap_v13.1.29/Design/online_designer.php?pid=5429&page=dashboard_features_and_indicators&field=other_software&branching=1) | 47 | [other_software]  Show the field ONLY if:  [dash_software] = '3' | Other dashboard software | text, Required Question number: 22 |
| [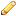](https://base.uams.edu/redcap/redcap_v13.1.29/Design/online_designer.php?pid=5429&page=dashboard_features_and_indicators&field=health_status)  [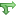](https://base.uams.edu/redcap/redcap_v13.1.29/Design/online_designer.php?pid=5429&page=dashboard_features_and_indicators&field=health_status&branching=1) | 48 | [health_status] | Section Header: *Section 2: Indicators and Reporting Format among Reviewed Maternal Health Dashboards A) HEALTH STATUS*  Does the dashboard have any indicators on Health Status? | yesno, Required   \| 1 \| Yes \| \| --- \| --- \| \| 0 \| No \|   Custom alignment: LV Question number: 23 |
| [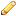](https://base.uams.edu/redcap/redcap_v13.1.29/Design/online_designer.php?pid=5429&page=dashboard_features_and_indicators&field=health_status_table)  [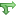](https://base.uams.edu/redcap/redcap_v13.1.29/Design/online_designer.php?pid=5429&page=dashboard_features_and_indicators&field=health_status_table&branching=1) | 49 | [health_status_table]  Show the field ONLY if:  [health_status] = '1' | Category Any Indicator Format Time period Geography Notes Births {births} {births_format} {births_time_period} {births_geo_agg} {hs_notes_b} Maternal Mortality {maternal_mortality} {mm_format} {mm_period} {mm_geo_agg} {hs_notes_mm} Infant Mortality {neonatal_mortality} {nm_format} {nm_period} {nm_geo_agg} {hs_notes_nm} Severe Maternal Morbidity {maternal_morbidity} {smm_format} {smm_period} {smm_geo_agg} {hs_notes_smm} Preterm Birth {preterm_birth} {pb_format} {pb_period} {pb_geo_agg} {hs_notes_pb} Birthweight {birthweight} {bw_format} {bw_period} {bw_geo_agg} {hs_notes_bw} Mode of Delivery {mode_of_delivery} {mod_format} {mod_period} {mod_geo_agg} {hs_notes_mod} Neonatal Withdrawal (NAS) {neonatal_withdrawal} {nas_format} {nas_period} {nas_geo_agg} {hs_notes_nas} Maternal Depression/Anxiety {maternal_depression} {mda_format} {mda_period} {mda_geo_agg} {hs_notes_mda} Pregnancy complications {gh} {gh_format} {gh_period} {gh_geo_agg} {gh_notes} Birth defects, congenital anomalies, abnormal infant outcomes {bd} {bd_format} {bd_period} {bd_geo_agg} {bd_notes} Other (please specify) {other_hs_category} {other_hs_exists} {other_hs_format} {other_hs_period} {other_hs_geo_agg} {hs_notes_other} | descriptive |
| [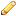](https://base.uams.edu/redcap/redcap_v13.1.29/Design/online_designer.php?pid=5429&page=dashboard_features_and_indicators&field=gh)  [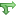](https://base.uams.edu/redcap/redcap_v13.1.29/Design/online_designer.php?pid=5429&page=dashboard_features_and_indicators&field=gh&branching=1) | 50 | [gh]  Show the field ONLY if:  [health_status] =1 |  | yesno   \| 1 \| Yes \| \| --- \| --- \| \| 0 \| No \| |
| [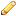](https://base.uams.edu/redcap/redcap_v13.1.29/Design/online_designer.php?pid=5429&page=dashboard_features_and_indicators&field=bd) | 51 | [bd]  Show the field ONLY if:  [health_status]=1 |  | yesno   \| 1 \| Yes \| \| --- \| --- \| \| 0 \| No \| |
|  | 52 | [births]  Show the field ONLY if:  [health_status]='1' | Birth data exists | yesno, Required   \| 1 \| Yes \| \| --- \| --- \| \| 0 \| No \|   Question number: 25 |
|  | 53 | [maternal_mortality]  Show the field ONLY if:  [health_status]='1' | maternal mortality data exists | yesno, Required   \| 1 \| Yes \| \| --- \| --- \| \| 0 \| No \|   Question number: 26 |
|  | 54 | [neonatal_mortality]  Show the field ONLY if:  [health_status]='1' | Infant mortality data exists | yesno, Required   \| 1 \| Yes \| \| --- \| --- \| \| 0 \| No \|   Question number: 27 |
|  | 55 | [other_hs_category]  Show the field ONLY if:  [other_hs_exists] = '1' | Other category of health status data | text, Required Custom alignment: LV Question number: 24 |
|  | 56 | [maternal_morbidity]  Show the field ONLY if:  [health_status]='1' | Severe maternal morbidity data exists | yesno, Required   \| 1 \| Yes \| \| --- \| --- \| \| 0 \| No \|   Question number: 28 |
|  | 57 | [preterm_birth]  Show the field ONLY if:  [health_status]='1' | preterm birth data exists | yesno, Required   \| 1 \| Yes \| \| --- \| --- \| \| 0 \| No \|   Question number: 29 |
|  | 58 | [birthweight]  Show the field ONLY if:  [health_status]='1' | birthweight data exists | yesno, Required   \| 1 \| Yes \| \| --- \| --- \| \| 0 \| No \|   Question number: 30 |
|  | 59 | [mode_of_delivery]  Show the field ONLY if:  [health_status]='1' | mode of delivery data exists | yesno, Required   \| 1 \| Yes \| \| --- \| --- \| \| 0 \| No \|   Question number: 31 |
|  | 60 | [neonatal_withdrawal]  Show the field ONLY if:  [health_status]='1' | neonatal withdrawal data exists | yesno, Required   \| 1 \| Yes \| \| --- \| --- \| \| 0 \| No \|   Question number: 32 |
|  | 61 | [maternal_depression]  Show the field ONLY if:  [health_status]='1' | Maternal depression/anxiety data exists | yesno, Required   \| 1 \| Yes \| \| --- \| --- \| \| 0 \| No \|   Question number: 33 |
|  | 62 | [other_hs_exists]  Show the field ONLY if:  [health_status]='1' | Other health status data exists | yesno, Required   \| 1 \| Yes \| \| --- \| --- \| \| 0 \| No \|   Question number: 34 |
|  | 63 | [births_format]  Show the field ONLY if:  [births] = '1' | birth data format | checkbox, Required   \| 1 \| births_format___1 \| Numbers \| \| --- \| --- \| --- \| \| 2 \| births_format___2 \| Rates/% \| \| 99 \| births_format___99 \| Other (specify in notes) \|   Custom alignment: LV Question number: 35 |
|  | 64 | [gh_format]  Show the field ONLY if:  [gh] = '1' |  | checkbox, Required   \| 1 \| gh_format___1 \| Numbers \| \| --- \| --- \| --- \| \| 2 \| gh_format___2 \| Rates/% \| \| 99 \| gh_format___99 \| Other (specify in notes) \|   Custom alignment: LV Question number: 35 |
|  | 65 | [bd_format]  Show the field ONLY if:  [bd] = '1' | birth data format | checkbox, Required   \| 1 \| bd_format___1 \| Numbers \| \| --- \| --- \| --- \| \| 2 \| bd_format___2 \| Rates/% \| \| 99 \| bd_format___99 \| Other (specify in notes) \|   Custom alignment: LV Question number: 35 |
|  | 66 | [mm_format]  Show the field ONLY if:  [maternal_mortality] = '1' | maternal mortality data format | checkbox, Required   \| 1 \| mm_format___1 \| Numbers \| \| --- \| --- \| --- \| \| 2 \| mm_format___2 \| Rates/% \| \| 99 \| mm_format___99 \| Other (specify in notes) \|   Custom alignment: LV Question number: 36 |
|  | 67 | [nm_format]  Show the field ONLY if:  [neonatal_mortality] = '1' | neonatal mortality data format | checkbox, Required   \| 1 \| nm_format___1 \| Numbers \| \| --- \| --- \| --- \| \| 2 \| nm_format___2 \| Rates/% \| \| 99 \| nm_format___99 \| Other (specify in notes) \|   Custom alignment: LV Question number: 37 |
|  | 68 | [smm_format]  Show the field ONLY if:  [maternal_morbidity] = '1' | Severe maternal morbidity data format | checkbox, Required   \| 1 \| smm_format___1 \| Numbers \| \| --- \| --- \| --- \| \| 2 \| smm_format___2 \| Rates/% \| \| 99 \| smm_format___99 \| Other (specify in notes) \|   Custom alignment: LV Question number: 38 |
|  | 69 | [pb_format]  Show the field ONLY if:  [preterm_birth] = '1' | preterm birth data format | checkbox, Required   \| 1 \| pb_format___1 \| Numbers \| \| --- \| --- \| --- \| \| 2 \| pb_format___2 \| Rates/% \| \| 99 \| pb_format___99 \| Other (specify in notes) \|   Custom alignment: LV Question number: 39 |
|  | 70 | [bw_format]  Show the field ONLY if:  [birthweight] = '1' | birthweight data format | checkbox, Required   \| 1 \| bw_format___1 \| Numbers \| \| --- \| --- \| --- \| \| 2 \| bw_format___2 \| Rates/% \| \| 99 \| bw_format___99 \| Other (specify in notes) \|   Custom alignment: LV Question number: 40 |
|  | 71 | [mod_format]  Show the field ONLY if:  [mode_of_delivery] = '1' | mode of delivery data format | checkbox, Required   \| 1 \| mod_format___1 \| Numbers \| \| --- \| --- \| --- \| \| 2 \| mod_format___2 \| Rates/% \| \| 99 \| mod_format___99 \| Other (specify in notes) \|   Custom alignment: LV Question number: 41 |
|  | 72 | [nas_format]  Show the field ONLY if:  [neonatal_withdrawal] = '1' | neonatal withdrawal data format | checkbox, Required   \| 1 \| nas_format___1 \| Numbers \| \| --- \| --- \| --- \| \| 2 \| nas_format___2 \| Rates/% \| \| 99 \| nas_format___99 \| Other (specify in notes) \|   Custom alignment: LV Question number: 42 |
|  | 73 | [mda_format]  Show the field ONLY if:  [maternal_depression] = '1' | maternal depression/anxiety data format | checkbox, Required   \| 1 \| mda_format___1 \| Numbers \| \| --- \| --- \| --- \| \| 2 \| mda_format___2 \| Rates/% \| \| 99 \| mda_format___99 \| Other (specify in notes) \|   Custom alignment: LV Question number: 43 |
|  | 74 | [other_hs_format]  Show the field ONLY if:  [other_hs_exists] = '1' | other data format | checkbox, Required   \| 1 \| other_hs_format___1 \| Numbers \| \| --- \| --- \| --- \| \| 2 \| other_hs_format___2 \| Rates/% \| \| 99 \| other_hs_format___99 \| Other (specify in notes) \|   Custom alignment: LV Question number: 44 |
|  | 75 | [births_time_period]  Show the field ONLY if:  [births] = '1' | Birth reporting time period | checkbox, Required   \| 1 \| births_time_period___1 \| Annual \| \| --- \| --- \| --- \| \| 2 \| births_time_period___2 \| Quarterly \| \| 3 \| births_time_period___3 \| Monthly \| \| 99 \| births_time_period___99 \| Other (specify in notes) \|   Custom alignment: LV Question number: 45 |
|  | 76 | [gh_period]  Show the field ONLY if:  [gh] = '1' |  | checkbox, Required   \| 1 \| gh_period___1 \| Annual \| \| --- \| --- \| --- \| \| 2 \| gh_period___2 \| Quarterly \| \| 3 \| gh_period___3 \| Monthly \| \| 99 \| gh_period___99 \| Other (specify in notes) \|   Custom alignment: LV Question number: 45 |
|  | 77 | [bd_period]  Show the field ONLY if:  [bd] = '1' |  | checkbox, Required   \| 1 \| bd_period___1 \| Annual \| \| --- \| --- \| --- \| \| 2 \| bd_period___2 \| Quarterly \| \| 3 \| bd_period___3 \| Monthly \| \| 99 \| bd_period___99 \| Other (specify in notes) \|   Custom alignment: LV Question number: 45 |
|  | 78 | [mm_period]  Show the field ONLY if:  [maternal_mortality] = '1' | Maternal mortality reporting time period | checkbox, Required   \| 1 \| mm_period___1 \| Annual \| \| --- \| --- \| --- \| \| 2 \| mm_period___2 \| Quarterly \| \| 3 \| mm_period___3 \| Monthly \| \| 99 \| mm_period___99 \| Other (specify in notes) \|   Custom alignment: LV Question number: 46 |
|  | 79 | [nm_period]  Show the field ONLY if:  [neonatal_mortality] = '1' | neonatal mortality reporting time period | checkbox, Required   \| 1 \| nm_period___1 \| Annual \| \| --- \| --- \| --- \| \| 2 \| nm_period___2 \| Quarterly \| \| 3 \| nm_period___3 \| Monthly \| \| 99 \| nm_period___99 \| Other (specify in notes) \|   Custom alignment: LV Question number: 47 |
|  | 80 | [smm_period]  Show the field ONLY if:  [maternal_morbidity] = '1' | severe maternal morbidity reporting time period | checkbox, Required   \| 1 \| smm_period___1 \| Annual \| \| --- \| --- \| --- \| \| 2 \| smm_period___2 \| Quarterly \| \| 3 \| smm_period___3 \| Monthly \| \| 99 \| smm_period___99 \| Other (specify in notes) \|   Custom alignment: LV Question number: 48 |
|  | 81 | [pb_period]  Show the field ONLY if:  [preterm_birth] = '1' | Preterm Birth reporting time period | checkbox, Required   \| 1 \| pb_period___1 \| Annual \| \| --- \| --- \| --- \| \| 2 \| pb_period___2 \| Quarterly \| \| 3 \| pb_period___3 \| Monthly \| \| 99 \| pb_period___99 \| Other (specify in notes) \|   Custom alignment: LV Question number: 49 |
|  | 82 | [bw_period]  Show the field ONLY if:  [birthweight] = '1' | Birthweight reporting time period | checkbox, Required   \| 1 \| bw_period___1 \| Annual \| \| --- \| --- \| --- \| \| 2 \| bw_period___2 \| Quarterly \| \| 3 \| bw_period___3 \| Monthly \| \| 99 \| bw_period___99 \| Other (specify in notes) \|   Custom alignment: LV Question number: 50 |
|  | 83 | [mod_period]  Show the field ONLY if:  [mode_of_delivery] = '1' | mode of delivery reporting time period | checkbox, Required   \| 1 \| mod_period___1 \| Annual \| \| --- \| --- \| --- \| \| 2 \| mod_period___2 \| Quarterly \| \| 3 \| mod_period___3 \| Monthly \| \| 99 \| mod_period___99 \| Other (specify in notes) \|   Custom alignment: LV Question number: 51 |
|  | 84 | [nas_period]  Show the field ONLY if:  [neonatal_withdrawal] = '1' | neonatal withdrawal reporting time period | checkbox, Required   \| 1 \| nas_period___1 \| Annual \| \| --- \| --- \| --- \| \| 2 \| nas_period___2 \| Quarterly \| \| 3 \| nas_period___3 \| Monthly \| \| 99 \| nas_period___99 \| Other (specify in notes) \|   Custom alignment: LV Question number: 52 |
|  | 85 | [mda_period]  Show the field ONLY if:  [maternal_depression] = '1' | maternal depression/anxiety reporting time period | checkbox, Required   \| 1 \| mda_period___1 \| Annual \| \| --- \| --- \| --- \| \| 2 \| mda_period___2 \| Quarterly \| \| 3 \| mda_period___3 \| Monthly \| \| 99 \| mda_period___99 \| Other (specify in notes) \|   Custom alignment: LV Question number: 53 |
|  | 86 | [other_hs_period]  Show the field ONLY if:  [other_hs_exists] = '1' | Other data reporting time period | checkbox, Required   \| 1 \| other_hs_period___1 \| Annual \| \| --- \| --- \| --- \| \| 2 \| other_hs_period___2 \| Quarterly \| \| 3 \| other_hs_period___3 \| Monthly \| \| 99 \| other_hs_period___99 \| Other (specify in notes) \|   Custom alignment: LV Question number: 54 |
|  | 87 | [births_geo_agg]  Show the field ONLY if:  [births] = '1' | Birth geographical aggregation | checkbox, Required   \| 1 \| births_geo_agg___1 \| National \| \| --- \| --- \| --- \| \| 2 \| births_geo_agg___2 \| Regional \| \| 3 \| births_geo_agg___3 \| State \| \| 4 \| births_geo_agg___4 \| State Region \| \| 5 \| births_geo_agg___5 \| County \| \| 6 \| births_geo_agg___6 \| Smaller than county (specify) \|   Custom alignment: LV Question number: 55 |
|  | 88 | [gh_geo_agg]  Show the field ONLY if:  [gh] = '1' | Birth geographical aggregation | checkbox, Required   \| 1 \| gh_geo_agg___1 \| National \| \| --- \| --- \| --- \| \| 2 \| gh_geo_agg___2 \| Regional \| \| 3 \| gh_geo_agg___3 \| State \| \| 4 \| gh_geo_agg___4 \| State Region \| \| 5 \| gh_geo_agg___5 \| County \| \| 6 \| gh_geo_agg___6 \| Smaller than county (specify) \|   Custom alignment: LV Question number: 55 |
|  | 89 | [bd_geo_agg]  Show the field ONLY if:  [bd] = '1' |  | checkbox, Required   \| 1 \| bd_geo_agg___1 \| National \| \| --- \| --- \| --- \| \| 2 \| bd_geo_agg___2 \| Regional \| \| 3 \| bd_geo_agg___3 \| State \| \| 4 \| bd_geo_agg___4 \| State Region \| \| 5 \| bd_geo_agg___5 \| County \| \| 6 \| bd_geo_agg___6 \| Smaller than county (specify) \|   Custom alignment: LV Question number: 55 |
|  | 90 | [mm_geo_agg]  Show the field ONLY if:  [maternal_mortality] = '1' | Maternal mortality geographical aggregation | checkbox, Required   \| 1 \| mm_geo_agg___1 \| National \| \| --- \| --- \| --- \| \| 2 \| mm_geo_agg___2 \| Regional \| \| 3 \| mm_geo_agg___3 \| State \| \| 4 \| mm_geo_agg___4 \| State Region \| \| 5 \| mm_geo_agg___5 \| County \| \| 6 \| mm_geo_agg___6 \| Smaller than county (specify) \|   Custom alignment: LV Question number: 56 |
|  | 91 | [nm_geo_agg]  Show the field ONLY if:  [neonatal_mortality] = '1' | neonatal mortality geographical aggregation | checkbox, Required   \| 1 \| nm_geo_agg___1 \| National \| \| --- \| --- \| --- \| \| 2 \| nm_geo_agg___2 \| Regional \| \| 3 \| nm_geo_agg___3 \| State \| \| 4 \| nm_geo_agg___4 \| State Region \| \| 5 \| nm_geo_agg___5 \| County \| \| 6 \| nm_geo_agg___6 \| Smaller than county (specify) \|   Custom alignment: LV Question number: 57 |
|  | 92 | [smm_geo_agg]  Show the field ONLY if:  [maternal_morbidity] = '1' | severe maternal morbidity geographical aggregation | checkbox, Required   \| 1 \| smm_geo_agg___1 \| National \| \| --- \| --- \| --- \| \| 2 \| smm_geo_agg___2 \| Regional \| \| 3 \| smm_geo_agg___3 \| State \| \| 4 \| smm_geo_agg___4 \| State Region \| \| 5 \| smm_geo_agg___5 \| County \| \| 6 \| smm_geo_agg___6 \| Smaller than county (specify) \|   Custom alignment: LV Question number: 58 |
|  | 93 | [pb_geo_agg]  Show the field ONLY if:  [preterm_birth] = '1' | Preterm Birth geographical aggregation | checkbox, Required   \| 1 \| pb_geo_agg___1 \| National \| \| --- \| --- \| --- \| \| 2 \| pb_geo_agg___2 \| Regional \| \| 3 \| pb_geo_agg___3 \| State \| \| 4 \| pb_geo_agg___4 \| State Region \| \| 5 \| pb_geo_agg___5 \| County \| \| 6 \| pb_geo_agg___6 \| Smaller than county (specify) \|   Custom alignment: LV Question number: 59 |
|  | 94 | [bw_geo_agg]  Show the field ONLY if:  [birthweight] = '1' | Birthweight geographical aggregation | checkbox, Required   \| 1 \| bw_geo_agg___1 \| National \| \| --- \| --- \| --- \| \| 2 \| bw_geo_agg___2 \| Regional \| \| 3 \| bw_geo_agg___3 \| State \| \| 4 \| bw_geo_agg___4 \| State Region \| \| 5 \| bw_geo_agg___5 \| County \| \| 6 \| bw_geo_agg___6 \| Smaller than county (specify) \|   Custom alignment: LV Question number: 60 |
|  | 95 | [mod_geo_agg]  Show the field ONLY if:  [mode_of_delivery] = '1' | Mode of delivery geographical aggregation | checkbox, Required   \| 1 \| mod_geo_agg___1 \| National \| \| --- \| --- \| --- \| \| 2 \| mod_geo_agg___2 \| Regional \| \| 3 \| mod_geo_agg___3 \| State \| \| 4 \| mod_geo_agg___4 \| State Region \| \| 5 \| mod_geo_agg___5 \| County \| \| 6 \| mod_geo_agg___6 \| Smaller than county (specify) \|   Custom alignment: LV Question number: 61 |
|  | 96 | [nas_geo_agg]  Show the field ONLY if:  [neonatal_withdrawal] = '1' | Neonatal withdrawal geographical aggregation | checkbox, Required   \| 1 \| nas_geo_agg___1 \| National \| \| --- \| --- \| --- \| \| 2 \| nas_geo_agg___2 \| Regional \| \| 3 \| nas_geo_agg___3 \| State \| \| 4 \| nas_geo_agg___4 \| State Region \| \| 5 \| nas_geo_agg___5 \| County \| \| 6 \| nas_geo_agg___6 \| Smaller than county (specify) \|   Custom alignment: LV Question number: 62 |
|  | 97 | [mda_geo_agg]  Show the field ONLY if:  [maternal_depression] = '1' | maternal anxiety/depression geographical aggregation | checkbox, Required   \| 1 \| mda_geo_agg___1 \| National \| \| --- \| --- \| --- \| \| 2 \| mda_geo_agg___2 \| Regional \| \| 3 \| mda_geo_agg___3 \| State \| \| 4 \| mda_geo_agg___4 \| State Region \| \| 5 \| mda_geo_agg___5 \| County \| \| 6 \| mda_geo_agg___6 \| Smaller than county (specify) \|   Custom alignment: LV Question number: 63 |
|  | 98 | [other_hs_geo_agg]  Show the field ONLY if:  [other_hs_exists] = '1' | other data geographical aggregation | checkbox, Required   \| 1 \| other_hs_geo_agg___1 \| National \| \| --- \| --- \| --- \| \| 2 \| other_hs_geo_agg___2 \| Regional \| \| 3 \| other_hs_geo_agg___3 \| State \| \| 4 \| other_hs_geo_agg___4 \| State Region \| \| 5 \| other_hs_geo_agg___5 \| County \| \| 6 \| other_hs_geo_agg___6 \| Smaller than county (specify) \|   Custom alignment: LV Question number: 64 |
|  | 99 | [hs_notes_b]  Show the field ONLY if:  [births]='1' | Births notes | notes |
|  | 100 | [gh_notes]  Show the field ONLY if:  [gh]='1' |  | notes |
|  | 101 | [bd_notes]  Show the field ONLY if:  [bd]='1' |  | notes |
|  | 102 | [hs_notes_mm]  Show the field ONLY if:  [maternal_mortality]='1' | Maternal mortality notes | notes |
|  | 103 | [hs_notes_nm]  Show the field ONLY if:  [neonatal_mortality]='1' | Neonatal mortality notes | notes |
|  | 104 | [hs_notes_smm]  Show the field ONLY if:  [maternal_morbidity]='1' | Severe maternal morbidity notes | notes |
|  | 105 | [hs_notes_pb]  Show the field ONLY if:  [preterm_birth]='1' | Preterm Birth notes | notes |
|  | 106 | [hs_notes_bw]  Show the field ONLY if:  [birthweight]='1' | Birthweight notes | notes |
|  | 107 | [hs_notes_mod]  Show the field ONLY if:  [mode_of_delivery]='1' | Mode of delivery notes | notes |
|  | 108 | [hs_notes_nas]  Show the field ONLY if:  [neonatal_withdrawal]='1' | Neonatal withdrawal notes | notes |
|  | 109 | [hs_notes_mda]  Show the field ONLY if:  [maternal_depression]='1' | Maternal depression/anxiety notes | notes |
|  | 110 | [hs_notes_other]  Show the field ONLY if:  [other_hs_exists]='1' | Other Health Status indicator notes | notes |
|  | 111 | [health_behaviours_careseeking] | Section Header: *Section 2: Indicators and Reporting Format among Reviewed Maternal Health Dashboards B) HEALTH BEHAVIOURS AND UTILIZATION*  Does the dashboard have any indicators on Health Behaviours and Utilization? | yesno, Required   \| 1 \| Yes \| \| --- \| --- \| \| 0 \| No \|   Custom alignment: LV Question number: 65 |
|  | 112 | [hbc_table]  Show the field ONLY if:  [health_behaviours_careseeking] = '1' | Category Any Indicator Format Time period Geography Notes Preventative care {hbc_prev} {hbc_format_prev} {hbc_period_prev} {hbc_geo_agg_prev} {hbc_notes_prev} Preconception care {hbc_prec} {hbc_format_prec} {hbc_period_prec} {hbc_geo_agg_prec} {hbc_notes_prec} Prenatal care {hbc_pn} {hbc_format_pn} {hbc_period_pn} {hbc_geo_agg_pn} {hbc_notes_pn} Postpartum care {hbc_post} {hbc_format_post} {hbc_period_post} {hbc_geo_agg_post} {hbc_notes_post} Perinatal Oral Health {hbc_oh} {hbc_format_oh} {hbc_period_oh} {hbc_geo_agg_oh} {hbc_notes_oh} Substance use in pregnancy {hbc_su} {hbc_format_su} {hbc_period_su} {hbc_geo_agg_su} {hbc_notes_su} Maternal Smoking {hbc_ms} {hbc_format_ms} {hbc_period_ms} {hbc_geo_agg_ms} {hbc_notes_ms} Maternal Nutrition {hbc_mn} {hbc_format_mn} {hbc_period_mn} {hbc_geo_agg_mn} {hbc_notes_mn} Breastfeeding {hbc_b} {hbc_format_b} {hbc_period_b} {hbc_geo_agg_b} {hbc_notes_b} Immunization {hbc_i} {hbc_format_i} {hbc_period_i} {hbc_geo_agg_i} {hbc_notes_i} Other (please specify) {other_hbc_category} {other_hbc_exists} {other_hbc_format} {other_hbc_period} {other_hbc_geo_agg} {hbc_notes_other} | descriptive |
|  | 113 | [other_hbc_category]  Show the field ONLY if:  [other_hbc_exists]='1' | Other hbc category | text, Required Custom alignment: LV Question number: 66 |
|  | 114 | [hbc_prev]  Show the field ONLY if:  [health_behaviours_careseeking]='1' |  | yesno, Required   \| 1 \| Yes \| \| --- \| --- \| \| 0 \| No \|   Custom alignment: LV Question number: 67 |
|  | 115 | [hbc_prec]  Show the field ONLY if:  [health_behaviours_careseeking]='1' |  | yesno, Required   \| 1 \| Yes \| \| --- \| --- \| \| 0 \| No \|   Custom alignment: LV Question number: 67 |
|  | 116 | [hbc_post]  Show the field ONLY if:  [health_behaviours_careseeking]='1' |  | yesno, Required   \| 1 \| Yes \| \| --- \| --- \| \| 0 \| No \|   Custom alignment: LV Question number: 67 |
|  | 117 | [hbc_b]  Show the field ONLY if:  [health_behaviours_careseeking]='1' |  | yesno, Required   \| 1 \| Yes \| \| --- \| --- \| \| 0 \| No \|   Custom alignment: LV Question number: 67 |
|  | 118 | [hbc_i]  Show the field ONLY if:  [health_behaviours_careseeking]='1' |  | yesno, Required   \| 1 \| Yes \| \| --- \| --- \| \| 0 \| No \|   Custom alignment: LV Question number: 67 |
|  | 119 | [hbc_pn]  Show the field ONLY if:  [health_behaviours_careseeking]='1' | prenatal care data exists | yesno, Required   \| 1 \| Yes \| \| --- \| --- \| \| 0 \| No \|   Custom alignment: LV Question number: 67 |
|  | 120 | [hbc_oh]  Show the field ONLY if:  [health_behaviours_careseeking]='1' | perinatal oral health data exists | yesno, Required   \| 1 \| Yes \| \| --- \| --- \| \| 0 \| No \|   Custom alignment: LV Question number: 68 |
|  | 121 | [hbc_su]  Show the field ONLY if:  [health_behaviours_careseeking]='1' | substance use in pregnancy data exists | yesno, Required   \| 1 \| Yes \| \| --- \| --- \| \| 0 \| No \|   Custom alignment: LV Question number: 69 |
|  | 122 | [hbc_ms]  Show the field ONLY if:  [health_behaviours_careseeking]='1' | maternal smoking data exists | yesno, Required   \| 1 \| Yes \| \| --- \| --- \| \| 0 \| No \|   Custom alignment: LV Question number: 70 |
|  | 123 | [hbc_mn]  Show the field ONLY if:  [health_behaviours_careseeking]='1' | maternal nutrition data exists | yesno, Required   \| 1 \| Yes \| \| --- \| --- \| \| 0 \| No \|   Custom alignment: LV Question number: 71 |
|  | 124 | [other_hbc_exists]  Show the field ONLY if:  [health_behaviours_careseeking]='1' | other hbc data exists | yesno, Required   \| 1 \| Yes \| \| --- \| --- \| \| 0 \| No \|   Custom alignment: LV Question number: 72 |
|  | 125 | [hbc_format_prev]  Show the field ONLY if:  [hbc_prev]='1' |  | checkbox, Required   \| 1 \| hbc_format_prev___1 \| Numbers \| \| --- \| --- \| --- \| \| 2 \| hbc_format_prev___2 \| Rates, % \| \| 99 \| hbc_format_prev___99 \| Other (specify in notes) \|   Custom alignment: LV Question number: 73 |
|  | 126 | [hbc_format_prec]  Show the field ONLY if:  [hbc_prec]='1' |  | checkbox, Required   \| 1 \| hbc_format_prec___1 \| Numbers \| \| --- \| --- \| --- \| \| 2 \| hbc_format_prec___2 \| Rates, % \| \| 99 \| hbc_format_prec___99 \| Other (specify in notes) \|   Custom alignment: LV Question number: 73 |
|  | 127 | [hbc_format_post]  Show the field ONLY if:  [hbc_post]='1' |  | checkbox, Required   \| 1 \| hbc_format_post___1 \| Numbers \| \| --- \| --- \| --- \| \| 2 \| hbc_format_post___2 \| Rates, % \| \| 99 \| hbc_format_post___99 \| Other (specify in notes) \|   Custom alignment: LV Question number: 73 |
|  | 128 | [hbc_format_b]  Show the field ONLY if:  [hbc_b]='1' |  | checkbox, Required   \| 1 \| hbc_format_b___1 \| Numbers \| \| --- \| --- \| --- \| \| 2 \| hbc_format_b___2 \| Rates, % \| \| 99 \| hbc_format_b___99 \| Other (specify in notes) \|   Custom alignment: LV Question number: 73 |
|  | 129 | [hbc_format_i]  Show the field ONLY if:  [hbc_i]='1' |  | checkbox, Required   \| 1 \| hbc_format_i___1 \| Numbers \| \| --- \| --- \| --- \| \| 2 \| hbc_format_i___2 \| Rates, % \| \| 99 \| hbc_format_i___99 \| Other (specify in notes) \|   Custom alignment: LV Question number: 73 |
|  | 130 | [hbc_format_pn]  Show the field ONLY if:  [hbc_pn]='1' | Prenatal care data format | checkbox, Required   \| 1 \| hbc_format_pn___1 \| Numbers \| \| --- \| --- \| --- \| \| 2 \| hbc_format_pn___2 \| Rates, % \| \| 99 \| hbc_format_pn___99 \| Other (specify in notes) \|   Custom alignment: LV Question number: 73 |
|  | 131 | [hbc_format_oh]  Show the field ONLY if:  [hbc_oh] = '1' | Perinatal oral health data format | checkbox, Required   \| 1 \| hbc_format_oh___1 \| Numbers \| \| --- \| --- \| --- \| \| 2 \| hbc_format_oh___2 \| Rates, % \| \| 99 \| hbc_format_oh___99 \| Other (specify in notes) \|   Custom alignment: LV Question number: 74 |
|  | 132 | [hbc_format_su]  Show the field ONLY if:  [hbc_su]='1' | Substance use in pregnancy data format | checkbox, Required   \| 1 \| hbc_format_su___1 \| Numbers \| \| --- \| --- \| --- \| \| 2 \| hbc_format_su___2 \| Rates, % \| \| 99 \| hbc_format_su___99 \| Other (specify in notes) \|   Custom alignment: LV Question number: 75 |
|  | 133 | [hbc_format_ms]  Show the field ONLY if:  [hbc_ms]='1' | Maternal smoking data format | checkbox, Required   \| 1 \| hbc_format_ms___1 \| Numbers \| \| --- \| --- \| --- \| \| 2 \| hbc_format_ms___2 \| Rates, % \| \| 99 \| hbc_format_ms___99 \| Other (specify in notes) \|   Custom alignment: LV Question number: 76 |
|  | 134 | [hbc_format_mn]  Show the field ONLY if:  [hbc_mn]='1' | maternal nutrition data format | checkbox, Required   \| 1 \| hbc_format_mn___1 \| Numbers \| \| --- \| --- \| --- \| \| 2 \| hbc_format_mn___2 \| Rates, % \| \| 99 \| hbc_format_mn___99 \| Other (specify in notes) \|   Custom alignment: LV Question number: 77 |
|  | 135 | [other_hbc_format]  Show the field ONLY if:  [other_hbc_exists] = '1' | Other HBC data format | checkbox, Required   \| 1 \| other_hbc_format___1 \| Numbers \| \| --- \| --- \| --- \| \| 2 \| other_hbc_format___2 \| Rates, % \| \| 99 \| other_hbc_format___99 \| Other (specify in notes) \|   Custom alignment: LV Question number: 78 |
|  | 136 | [hbc_period_prev]  Show the field ONLY if:  [hbc_prev] = '1' |  | checkbox, Required   \| 1 \| hbc_period_prev___1 \| Annual \| \| --- \| --- \| --- \| \| 2 \| hbc_period_prev___2 \| Quarterly \| \| 3 \| hbc_period_prev___3 \| Monthly \| \| 99 \| hbc_period_prev___99 \| Other (specify in notes) \|   Custom alignment: LV Question number: 79 |
|  | 137 | [hbc_period_prec]  Show the field ONLY if:  [hbc_prec] = '1' |  | checkbox, Required   \| 1 \| hbc_period_prec___1 \| Annual \| \| --- \| --- \| --- \| \| 2 \| hbc_period_prec___2 \| Quarterly \| \| 3 \| hbc_period_prec___3 \| Monthly \| \| 99 \| hbc_period_prec___99 \| Other (specify in notes) \|   Custom alignment: LV Question number: 79 |
|  | 138 | [hbc_period_post]  Show the field ONLY if:  [hbc_post] = '1' |  | checkbox, Required   \| 1 \| hbc_period_post___1 \| Annual \| \| --- \| --- \| --- \| \| 2 \| hbc_period_post___2 \| Quarterly \| \| 3 \| hbc_period_post___3 \| Monthly \| \| 99 \| hbc_period_post___99 \| Other (specify in notes) \|   Custom alignment: LV Question number: 79 |
|  | 139 | [hbc_period_b]  Show the field ONLY if:  [hbc_b] = '1' |  | checkbox, Required   \| 1 \| hbc_period_b___1 \| Annual \| \| --- \| --- \| --- \| \| 2 \| hbc_period_b___2 \| Quarterly \| \| 3 \| hbc_period_b___3 \| Monthly \| \| 99 \| hbc_period_b___99 \| Other (specify in notes) \|   Custom alignment: LV Question number: 79 |
|  | 140 | [hbc_period_i]  Show the field ONLY if:  [hbc_i] = '1' |  | checkbox, Required   \| 1 \| hbc_period_i___1 \| Annual \| \| --- \| --- \| --- \| \| 2 \| hbc_period_i___2 \| Quarterly \| \| 3 \| hbc_period_i___3 \| Monthly \| \| 99 \| hbc_period_i___99 \| Other (specify in notes) \|   Custom alignment: LV Question number: 79 |
|  | 141 | [hbc_period_pn]  Show the field ONLY if:  [hbc_pn] = '1' | Prenatal care time period | checkbox, Required   \| 1 \| hbc_period_pn___1 \| Annual \| \| --- \| --- \| --- \| \| 2 \| hbc_period_pn___2 \| Quarterly \| \| 3 \| hbc_period_pn___3 \| Monthly \| \| 99 \| hbc_period_pn___99 \| Other (specify in notes) \|   Custom alignment: LV Question number: 79 |
|  | 142 | [hbc_period_oh]  Show the field ONLY if:  [hbc_oh] = '1' | Perinatal oral health time period | checkbox, Required   \| 1 \| hbc_period_oh___1 \| Annual \| \| --- \| --- \| --- \| \| 2 \| hbc_period_oh___2 \| Quarterly \| \| 3 \| hbc_period_oh___3 \| Monthly \| \| 99 \| hbc_period_oh___99 \| Other (specify in notes) \|   Custom alignment: LV Question number: 80 |
|  | 143 | [hbc_period_su]  Show the field ONLY if:  [hbc_su] = '1' | substance use in pregnancy time period | checkbox, Required   \| 1 \| hbc_period_su___1 \| Annual \| \| --- \| --- \| --- \| \| 2 \| hbc_period_su___2 \| Quarterly \| \| 3 \| hbc_period_su___3 \| Monthly \| \| 99 \| hbc_period_su___99 \| Other (specify in notes) \|   Custom alignment: LV Question number: 81 |
|  | 144 | [hbc_period_ms]  Show the field ONLY if:  [hbc_ms] = '1' | maternal smoking time period | checkbox, Required   \| 1 \| hbc_period_ms___1 \| Annual \| \| --- \| --- \| --- \| \| 2 \| hbc_period_ms___2 \| Quarterly \| \| 3 \| hbc_period_ms___3 \| Monthly \| \| 99 \| hbc_period_ms___99 \| Other (specify in notes) \|   Custom alignment: LV Question number: 82 |
|  | 145 | [hbc_period_mn]  Show the field ONLY if:  [hbc_mn] = '1' | Maternal nutrition time period | checkbox, Required   \| 1 \| hbc_period_mn___1 \| Annual \| \| --- \| --- \| --- \| \| 2 \| hbc_period_mn___2 \| Quarterly \| \| 3 \| hbc_period_mn___3 \| Monthly \| \| 99 \| hbc_period_mn___99 \| Other (specify in notes) \|   Custom alignment: LV Question number: 83 |
|  | 146 | [other_hbc_period]  Show the field ONLY if:  [other_hbc_exists] = '1' | other hbc time period | checkbox, Required   \| 1 \| other_hbc_period___1 \| Annual \| \| --- \| --- \| --- \| \| 2 \| other_hbc_period___2 \| Quarterly \| \| 3 \| other_hbc_period___3 \| Monthly \| \| 99 \| other_hbc_period___99 \| Other (specify in notes) \|   Custom alignment: LV Question number: 84 |
|  | 147 | [hbc_geo_agg_prev]  Show the field ONLY if:  [hbc_prev]='1' |  | checkbox, Required   \| 1 \| hbc_geo_agg_prev___1 \| National \| \| --- \| --- \| --- \| \| 2 \| hbc_geo_agg_prev___2 \| Regional \| \| 3 \| hbc_geo_agg_prev___3 \| State \| \| 4 \| hbc_geo_agg_prev___4 \| State Region \| \| 5 \| hbc_geo_agg_prev___5 \| County \| \| 6 \| hbc_geo_agg_prev___6 \| Smaller than county (specify) \|   Custom alignment: LV Question number: 85 |
|  | 148 | [hbc_geo_agg_prec]  Show the field ONLY if:  [hbc_prec]='1' |  | checkbox, Required   \| 1 \| hbc_geo_agg_prec___1 \| National \| \| --- \| --- \| --- \| \| 2 \| hbc_geo_agg_prec___2 \| Regional \| \| 3 \| hbc_geo_agg_prec___3 \| State \| \| 4 \| hbc_geo_agg_prec___4 \| State Region \| \| 5 \| hbc_geo_agg_prec___5 \| County \| \| 6 \| hbc_geo_agg_prec___6 \| Smaller than county (specify) \|   Custom alignment: LV Question number: 85 |
|  | 149 | [hbc_geo_agg_post]  Show the field ONLY if:  [hbc_post]='1' |  | checkbox, Required   \| 1 \| hbc_geo_agg_post___1 \| National \| \| --- \| --- \| --- \| \| 2 \| hbc_geo_agg_post___2 \| Regional \| \| 3 \| hbc_geo_agg_post___3 \| State \| \| 4 \| hbc_geo_agg_post___4 \| State Region \| \| 5 \| hbc_geo_agg_post___5 \| County \| \| 6 \| hbc_geo_agg_post___6 \| Smaller than county (specify) \|   Custom alignment: LV Question number: 85 |
|  | 150 | [hbc_geo_agg_b]  Show the field ONLY if:  [hbc_b]='1' |  | checkbox, Required   \| 1 \| hbc_geo_agg_b___1 \| National \| \| --- \| --- \| --- \| \| 2 \| hbc_geo_agg_b___2 \| Regional \| \| 3 \| hbc_geo_agg_b___3 \| State \| \| 4 \| hbc_geo_agg_b___4 \| State Region \| \| 5 \| hbc_geo_agg_b___5 \| County \| \| 6 \| hbc_geo_agg_b___6 \| Smaller than county (specify) \|   Custom alignment: LV Question number: 85 |
|  | 151 | [hbc_geo_agg_i]  Show the field ONLY if:  [hbc_i]='1' |  | checkbox, Required   \| 1 \| hbc_geo_agg_i___1 \| National \| \| --- \| --- \| --- \| \| 2 \| hbc_geo_agg_i___2 \| Regional \| \| 3 \| hbc_geo_agg_i___3 \| State \| \| 4 \| hbc_geo_agg_i___4 \| State Region \| \| 5 \| hbc_geo_agg_i___5 \| County \| \| 6 \| hbc_geo_agg_i___6 \| Smaller than county (specify) \|   Custom alignment: LV Question number: 85 |
|  | 152 | [hbc_geo_agg_pn]  Show the field ONLY if:  [hbc_pn]='1' | Prenatal care data geographical aggregation | checkbox, Required   \| 1 \| hbc_geo_agg_pn___1 \| National \| \| --- \| --- \| --- \| \| 2 \| hbc_geo_agg_pn___2 \| Regional \| \| 3 \| hbc_geo_agg_pn___3 \| State \| \| 4 \| hbc_geo_agg_pn___4 \| State Region \| \| 5 \| hbc_geo_agg_pn___5 \| County \| \| 6 \| hbc_geo_agg_pn___6 \| Smaller than county (specify) \|   Custom alignment: LV Question number: 85 |
|  | 153 | [hbc_geo_agg_oh]  Show the field ONLY if:  [hbc_oh]='1' | Perinatal oral health data geographical aggregation | checkbox, Required   \| 1 \| hbc_geo_agg_oh___1 \| National \| \| --- \| --- \| --- \| \| 2 \| hbc_geo_agg_oh___2 \| Regional \| \| 3 \| hbc_geo_agg_oh___3 \| State \| \| 4 \| hbc_geo_agg_oh___4 \| State Region \| \| 5 \| hbc_geo_agg_oh___5 \| County \| \| 6 \| hbc_geo_agg_oh___6 \| Smaller than county (specify) \|   Custom alignment: LV Question number: 86 |
|  | 154 | [hbc_geo_agg_su]  Show the field ONLY if:  [hbc_su]='1' | Substance use in pregnancy data geographical aggregation | checkbox, Required   \| 1 \| hbc_geo_agg_su___1 \| National \| \| --- \| --- \| --- \| \| 2 \| hbc_geo_agg_su___2 \| Regional \| \| 3 \| hbc_geo_agg_su___3 \| State \| \| 4 \| hbc_geo_agg_su___4 \| State Region \| \| 5 \| hbc_geo_agg_su___5 \| County \| \| 6 \| hbc_geo_agg_su___6 \| Smaller than county (specify) \|   Custom alignment: LV Question number: 87 |
|  | 155 | [hbc_geo_agg_ms]  Show the field ONLY if:  [hbc_ms]='1' | Maternal smoking data geographical aggregation | checkbox, Required   \| 1 \| hbc_geo_agg_ms___1 \| National \| \| --- \| --- \| --- \| \| 2 \| hbc_geo_agg_ms___2 \| Regional \| \| 3 \| hbc_geo_agg_ms___3 \| State \| \| 4 \| hbc_geo_agg_ms___4 \| State Region \| \| 5 \| hbc_geo_agg_ms___5 \| County \| \| 6 \| hbc_geo_agg_ms___6 \| Smaller than county (specify) \|   Custom alignment: LV Question number: 88 |
|  | 156 | [hbc_geo_agg_mn]  Show the field ONLY if:  [hbc_mn]='1' | Maternal nutrition data geographical aggregation | checkbox, Required   \| 1 \| hbc_geo_agg_mn___1 \| National \| \| --- \| --- \| --- \| \| 2 \| hbc_geo_agg_mn___2 \| Regional \| \| 3 \| hbc_geo_agg_mn___3 \| State \| \| 4 \| hbc_geo_agg_mn___4 \| State Region \| \| 5 \| hbc_geo_agg_mn___5 \| County \| \| 6 \| hbc_geo_agg_mn___6 \| Smaller than county (specify) \|   Custom alignment: LV Question number: 89 |
|  | 157 | [other_hbc_geo_agg]  Show the field ONLY if:  [other_hbc_exists]='1' | Prenatal care data geographical aggregation | checkbox, Required   \| 1 \| other_hbc_geo_agg___1 \| National \| \| --- \| --- \| --- \| \| 2 \| other_hbc_geo_agg___2 \| Regional \| \| 3 \| other_hbc_geo_agg___3 \| State \| \| 4 \| other_hbc_geo_agg___4 \| State Region \| \| 5 \| other_hbc_geo_agg___5 \| County \| \| 6 \| other_hbc_geo_agg___6 \| Smaller than county (specify) \|   Custom alignment: LV Question number: 90 |
|  | 158 | [hbc_notes_prev]  Show the field ONLY if:  [hbc_prev]='1' |  | notes |
|  | 159 | [hbc_notes_prec]  Show the field ONLY if:  [hbc_prec]='1' |  | notes |
|  | 160 | [hbc_notes_post]  Show the field ONLY if:  [hbc_post]='1' |  | notes |
|  | 161 | [hbc_notes_b]  Show the field ONLY if:  [hbc_b]='1' |  | notes |
|  | 162 | [hbc_notes_i]  Show the field ONLY if:  [hbc_i]='1' |  | notes |
|  | 163 | [hbc_notes_pn]  Show the field ONLY if:  [hbc_pn]='1' | Prenatal care notes | notes |
|  | 164 | [hbc_notes_oh]  Show the field ONLY if:  [hbc_oh]='1' | Perinatal oral health notes | notes |
|  | 165 | [hbc_notes_su]  Show the field ONLY if:  [hbc_su]='1' | Substance use in pregnancy notes | notes |
|  | 166 | [hbc_notes_ms]  Show the field ONLY if:  [hbc_ms]='1' | Maternal smoking notes | notes |
|  | 167 | [hbc_notes_mn]  Show the field ONLY if:  [hbc_mn]='1' | Maternal nutrition notes | notes |
|  | 168 | [hbc_notes_other]  Show the field ONLY if:  [other_hbc_exists]='1' | Other HBC notes | notes |
|  | 169 | [ind_char_risk] | Section Header: *Section 2: Indicators and Reporting Format among Reviewed Maternal Health Dashboards C) INDIVIDUAL CHARACTERISTICS AND RISK FACTORS*  Does the dashboard have any indicators on Individual Characteristics and Risk Factors? | yesno, Required   \| 1 \| Yes \| \| --- \| --- \| \| 0 \| No \|   Custom alignment: LV |
|  | 170 | [icrf_table]  Show the field ONLY if:  [ind_char_risk] = '1' | Category Any Indicator Format Time period Geography Notes Preconception health (comorbidities) {icrf_ph} {icrf_format_ph} {icrf_period_ph} {icrf_geo_agg_ph} {icrf_notes_ph} Maternal BMI {icrf_mbmi} {icrf_format_mbmi} {icrf_period_mbmi} {icrf_geo_agg_mbmi} {icrf_notes_mbmi} Health Insurance Status  {icrf_hi} {icrf_format_hi} {icrf_period_hi} {icrf_geo_agg_hi} {icrf_notes_hi} Maternal Age {icrf_ma} {icrf_format_ma} {icrf_period_ma} {icrf_geo_agg_ma} {icrf_notes_ma} Maternal Education {icrf_me} {icrf_format_me} {icrf_period_me} {icrf_geo_agg_me} {icrf_notes_me} Maternal Race/Ethnicity {icrf_re} {icrf_format_re} {icrf_period_re} {icrf_geo_agg_re} {icrf_notes_re} Maternal marital status {icrf_ms} {icrf_format_ms} {icrf_period_ms} {icrf_geo_agg_ms} {icrf_notes_ms} Birth spacing {icrf_bs} {icrf_format_bs} {icrf_period_bs} {icrf_geo_agg_bs} {icrf_notes_bs} Stress/abuse {icrf_abu} {icrf_format_abu} {icrf_period_abu} {icrf_geo_agg_abu} {icrf_notes_abu} Pregnancy intention {icrf_int} {icrf_format_int} {icrf_period_int} {icrf_geo_agg_int} {icrf_notes_int} Num prior births {icrf_npb} {icrf_format_npb} {icrf_period_npb} {icrf_geo_agg_npb} {icrf_notes_npb} Plurality {icrf_plu} {icrf_format_plu} {icrf_period_plu} {icrf_geo_agg_plu} {icrf_notes_plu} Other (please specify) {other_icrf_category} {other_icrf_exists} {other_icrf_format} {other_icrf_period} {other_icrf_geo_agg} {icrf_notes_other} | descriptive |
|  | 171 | [other_icrf_category]  Show the field ONLY if:  [other_icrf_exists]='1' | Other icrf category | text, Required |
|  | 172 | [icrf_ph]  Show the field ONLY if:  [ind_char_risk]='1' | Preconception health data exists | yesno, Required   \| 1 \| Yes \| \| --- \| --- \| \| 0 \| No \|   Custom alignment: LV |
|  | 173 | [icrf_mbmi]  Show the field ONLY if:  [ind_char_risk]='1' | maternal BMI data exists | yesno, Required   \| 1 \| Yes \| \| --- \| --- \| \| 0 \| No \|   Custom alignment: LV |
|  | 174 | [icrf_hi]  Show the field ONLY if:  [ind_char_risk]='1' | Health Insurance Status data exists | yesno, Required   \| 1 \| Yes \| \| --- \| --- \| \| 0 \| No \|   Custom alignment: LV |
|  | 175 | [icrf_ma]  Show the field ONLY if:  [ind_char_risk]='1' | Maternal age data exists | yesno, Required   \| 1 \| Yes \| \| --- \| --- \| \| 0 \| No \|   Custom alignment: LV |
|  | 176 | [other_icrf_exists]  Show the field ONLY if:  [ind_char_risk]='1' | Other icrf data exists | yesno, Required   \| 1 \| Yes \| \| --- \| --- \| \| 0 \| No \|   Custom alignment: LV |
|  | 177 | [icrf_format_ph]  Show the field ONLY if:  [icrf_ph]='1' | Preconception health data format | checkbox, Required   \| 1 \| icrf_format_ph___1 \| Numbers \| \| --- \| --- \| --- \| \| 2 \| icrf_format_ph___2 \| Rates/% \| \| 99 \| icrf_format_ph___99 \| Other (specify in notes) \|   Custom alignment: LV |
|  | 178 | [icrf_format_mbmi]  Show the field ONLY if:  [icrf_mbmi]='1' | Maternal BMI data format | checkbox, Required   \| 1 \| icrf_format_mbmi___1 \| Numbers \| \| --- \| --- \| --- \| \| 2 \| icrf_format_mbmi___2 \| Rates/% \| \| 99 \| icrf_format_mbmi___99 \| Other (specify in notes) \|   Custom alignment: LV |
|  | 179 | [icrf_format_hi]  Show the field ONLY if:  [icrf_hi]='1' | Health insurance status data format | checkbox, Required   \| 1 \| icrf_format_hi___1 \| Numbers \| \| --- \| --- \| --- \| \| 2 \| icrf_format_hi___2 \| Rates/% \| \| 99 \| icrf_format_hi___99 \| Other (specify in notes) \|   Custom alignment: LV |
|  | 180 | [icrf_format_ma]  Show the field ONLY if:  [icrf_ma]='1' | Maternal age data format | checkbox, Required   \| 1 \| icrf_format_ma___1 \| Numbers \| \| --- \| --- \| --- \| \| 2 \| icrf_format_ma___2 \| Rates/% \| \| 99 \| icrf_format_ma___99 \| Other (specify in notes) \|   Custom alignment: LV |
|  | 181 | [other_icrf_format]  Show the field ONLY if:  [other_icrf_exists]='1' | Other icrf data format | checkbox, Required   \| 1 \| other_icrf_format___1 \| Numbers \| \| --- \| --- \| --- \| \| 2 \| other_icrf_format___2 \| Rates/% \| \| 99 \| other_icrf_format___99 \| Other (specify in notes) \|   Custom alignment: LV |
|  | 182 | [icrf_period_ph]  Show the field ONLY if:  [icrf_ph]='1' | Preconception health data time period | checkbox, Required   \| 1 \| icrf_period_ph___1 \| Annual \| \| --- \| --- \| --- \| \| 2 \| icrf_period_ph___2 \| Quarterly \| \| 3 \| icrf_period_ph___3 \| Monthly \| \| 99 \| icrf_period_ph___99 \| Other (specify in notes) \|   Custom alignment: LV |
|  | 183 | [icrf_period_mbmi]  Show the field ONLY if:  [icrf_mbmi]='1' | Maternal BMI data time period | checkbox, Required   \| 1 \| icrf_period_mbmi___1 \| Annual \| \| --- \| --- \| --- \| \| 2 \| icrf_period_mbmi___2 \| Quarterly \| \| 3 \| icrf_period_mbmi___3 \| Monthly \| \| 99 \| icrf_period_mbmi___99 \| Other (specify in notes) \|   Custom alignment: LV |
|  | 184 | [icrf_period_hi]  Show the field ONLY if:  [icrf_hi]='1' | Health Insurance Status data time period | checkbox, Required   \| 1 \| icrf_period_hi___1 \| Annual \| \| --- \| --- \| --- \| \| 2 \| icrf_period_hi___2 \| Quarterly \| \| 3 \| icrf_period_hi___3 \| Monthly \| \| 99 \| icrf_period_hi___99 \| Other (specify in notes) \|   Custom alignment: LV |
|  | 185 | [icrf_period_ma]  Show the field ONLY if:  [icrf_ma]='1' | Maternal age data time period | checkbox, Required   \| 1 \| icrf_period_ma___1 \| Annual \| \| --- \| --- \| --- \| \| 2 \| icrf_period_ma___2 \| Quarterly \| \| 3 \| icrf_period_ma___3 \| Monthly \| \| 99 \| icrf_period_ma___99 \| Other (specify in notes) \|   Custom alignment: LV |
|  | 186 | [other_icrf_period]  Show the field ONLY if:  [other_icrf_exists]='1' | Other icrf data time period | checkbox, Required   \| 1 \| other_icrf_period___1 \| Annual \| \| --- \| --- \| --- \| \| 2 \| other_icrf_period___2 \| Quarterly \| \| 3 \| other_icrf_period___3 \| Monthly \| \| 99 \| other_icrf_period___99 \| Other (specify in notes) \|   Custom alignment: LV |
|  | 187 | [icrf_geo_agg_ph]  Show the field ONLY if:  [icrf_ph]='1' | Preconception health data geographic aggregation | checkbox, Required   \| 1 \| icrf_geo_agg_ph___1 \| National \| \| --- \| --- \| --- \| \| 2 \| icrf_geo_agg_ph___2 \| Regional \| \| 3 \| icrf_geo_agg_ph___3 \| State \| \| 4 \| icrf_geo_agg_ph___4 \| State Region \| \| 5 \| icrf_geo_agg_ph___5 \| County \| \| 6 \| icrf_geo_agg_ph___6 \| Smaller than county (specify) \|   Custom alignment: LV |
|  | 188 | [icrf_geo_agg_mbmi]  Show the field ONLY if:  [icrf_mbmi]='1' | Maternal BMI data geographic aggregation | checkbox, Required   \| 1 \| icrf_geo_agg_mbmi___1 \| National \| \| --- \| --- \| --- \| \| 2 \| icrf_geo_agg_mbmi___2 \| Regional \| \| 3 \| icrf_geo_agg_mbmi___3 \| State \| \| 4 \| icrf_geo_agg_mbmi___4 \| State Region \| \| 5 \| icrf_geo_agg_mbmi___5 \| County \| \| 6 \| icrf_geo_agg_mbmi___6 \| Smaller than county (specify) \|   Custom alignment: LV |
|  | 189 | [icrf_geo_agg_hi]  Show the field ONLY if:  [icrf_hi]='1' | Health insurance status data geographic aggregation | checkbox, Required   \| 1 \| icrf_geo_agg_hi___1 \| National \| \| --- \| --- \| --- \| \| 2 \| icrf_geo_agg_hi___2 \| Regional \| \| 3 \| icrf_geo_agg_hi___3 \| State \| \| 4 \| icrf_geo_agg_hi___4 \| State Region \| \| 5 \| icrf_geo_agg_hi___5 \| County \| \| 6 \| icrf_geo_agg_hi___6 \| Smaller than county (specify) \|   Custom alignment: LV |
|  | 190 | [icrf_geo_agg_ma]  Show the field ONLY if:  [icrf_ma]='1' | Maternal age data geographic aggregation | checkbox, Required   \| 1 \| icrf_geo_agg_ma___1 \| National \| \| --- \| --- \| --- \| \| 2 \| icrf_geo_agg_ma___2 \| Regional \| \| 3 \| icrf_geo_agg_ma___3 \| State \| \| 4 \| icrf_geo_agg_ma___4 \| State Region \| \| 5 \| icrf_geo_agg_ma___5 \| County \| \| 6 \| icrf_geo_agg_ma___6 \| Smaller than county (specify) \|   Custom alignment: LV |
|  | 191 | [other_icrf_geo_agg]  Show the field ONLY if:  [other_icrf_exists]='1' | Other icrf data geographic aggregation | checkbox, Required   \| 1 \| other_icrf_geo_agg___1 \| National \| \| --- \| --- \| --- \| \| 2 \| other_icrf_geo_agg___2 \| Regional \| \| 3 \| other_icrf_geo_agg___3 \| State \| \| 4 \| other_icrf_geo_agg___4 \| State Region \| \| 5 \| other_icrf_geo_agg___5 \| County \| \| 6 \| other_icrf_geo_agg___6 \| Smaller than county (specify) \|   Custom alignment: LV |
|  | 192 | [icrf_notes_ph]  Show the field ONLY if:  [icrf_ph]='1' | Preconception health notes | notes |
|  | 193 | [icrf_notes_mbmi]  Show the field ONLY if:  [icrf_mbmi]='1' | Maternal BMI notes | notes |
|  | 194 | [icrf_notes_hi]  Show the field ONLY if:  [icrf_hi]='1' | Health insurance status notes | notes |
|  | 195 | [icrf_notes_ma]  Show the field ONLY if:  [icrf_ma]='1' | Maternal age notes | notes |
|  | 196 | [icrf_notes_other]  Show the field ONLY if:  [other_icrf_exists]='1' | Other ICRF indicator notes | notes |
|  | 197 | [icrf_me]  Show the field ONLY if:  [ind_char_risk]='1' |  | yesno, Required   \| 1 \| Yes \| \| --- \| --- \| \| 0 \| No \|   Custom alignment: LV |
|  | 198 | [icrf_re]  Show the field ONLY if:  [ind_char_risk]='1' |  | yesno, Required   \| 1 \| Yes \| \| --- \| --- \| \| 0 \| No \|   Custom alignment: LV |
|  | 199 | [icrf_ms]  Show the field ONLY if:  [ind_char_risk]='1' |  | yesno, Required   \| 1 \| Yes \| \| --- \| --- \| \| 0 \| No \|   Custom alignment: LV |
|  | 200 | [icrf_bs]  Show the field ONLY if:  [ind_char_risk]='1' |  | yesno, Required   \| 1 \| Yes \| \| --- \| --- \| \| 0 \| No \|   Custom alignment: LV |
|  | 201 | [icrf_abu]  Show the field ONLY if:  [ind_char_risk]='1' |  | yesno, Required   \| 1 \| Yes \| \| --- \| --- \| \| 0 \| No \|   Custom alignment: LV |
|  | 202 | [icrf_int]  Show the field ONLY if:  [ind_char_risk]='1' |  | yesno, Required   \| 1 \| Yes \| \| --- \| --- \| \| 0 \| No \|   Custom alignment: LV |
|  | 203 | [icrf_npb]  Show the field ONLY if:  [ind_char_risk]='1' |  | yesno, Required   \| 1 \| Yes \| \| --- \| --- \| \| 0 \| No \|   Custom alignment: LV |
|  | 204 | [icrf_plu]  Show the field ONLY if:  [ind_char_risk]='1' |  | yesno, Required   \| 1 \| Yes \| \| --- \| --- \| \| 0 \| No \|   Custom alignment: LV |
|  | 205 | [icrf_format_me]  Show the field ONLY if:  [icrf_me]='1' |  | checkbox, Required   \| 1 \| icrf_format_me___1 \| Numbers \| \| --- \| --- \| --- \| \| 2 \| icrf_format_me___2 \| Rates/% \| \| 99 \| icrf_format_me___99 \| Other (specify in notes) \|   Custom alignment: LV |
|  | 206 | [icrf_format_re]  Show the field ONLY if:  [icrf_re]='1' |  | checkbox, Required   \| 1 \| icrf_format_re___1 \| Numbers \| \| --- \| --- \| --- \| \| 2 \| icrf_format_re___2 \| Rates/% \| \| 99 \| icrf_format_re___99 \| Other (specify in notes) \|   Custom alignment: LV |
|  | 207 | [icrf_format_ms]  Show the field ONLY if:  [icrf_ms]='1' |  | checkbox, Required   \| 1 \| icrf_format_ms___1 \| Numbers \| \| --- \| --- \| --- \| \| 2 \| icrf_format_ms___2 \| Rates/% \| \| 99 \| icrf_format_ms___99 \| Other (specify in notes) \|   Custom alignment: LV |
|  | 208 | [icrf_format_bs]  Show the field ONLY if:  [icrf_bs]='1' |  | checkbox, Required   \| 1 \| icrf_format_bs___1 \| Numbers \| \| --- \| --- \| --- \| \| 2 \| icrf_format_bs___2 \| Rates/% \| \| 99 \| icrf_format_bs___99 \| Other (specify in notes) \|   Custom alignment: LV |
|  | 209 | [icrf_format_abu]  Show the field ONLY if:  [icrf_abu]='1' |  | checkbox, Required   \| 1 \| icrf_format_abu___1 \| Numbers \| \| --- \| --- \| --- \| \| 2 \| icrf_format_abu___2 \| Rates/% \| \| 99 \| icrf_format_abu___99 \| Other (specify in notes) \|   Custom alignment: LV |
|  | 210 | [icrf_format_int]  Show the field ONLY if:  [icrf_int]='1' |  | checkbox, Required   \| 1 \| icrf_format_int___1 \| Numbers \| \| --- \| --- \| --- \| \| 2 \| icrf_format_int___2 \| Rates/% \| \| 99 \| icrf_format_int___99 \| Other (specify in notes) \|   Custom alignment: LV |
|  | 211 | [icrf_format_npb]  Show the field ONLY if:  [icrf_npb]='1' |  | checkbox, Required   \| 1 \| icrf_format_npb___1 \| Numbers \| \| --- \| --- \| --- \| \| 2 \| icrf_format_npb___2 \| Rates/% \| \| 99 \| icrf_format_npb___99 \| Other (specify in notes) \|   Custom alignment: LV |
|  | 212 | [icrf_format_plu]  Show the field ONLY if:  [icrf_plu]='1' |  | checkbox, Required   \| 1 \| icrf_format_plu___1 \| Numbers \| \| --- \| --- \| --- \| \| 2 \| icrf_format_plu___2 \| Rates/% \| \| 99 \| icrf_format_plu___99 \| Other (specify in notes) \|   Custom alignment: LV |
|  | 213 | [icrf_period_me]  Show the field ONLY if:  [icrf_me]='1' |  | checkbox, Required   \| 1 \| icrf_period_me___1 \| Annual \| \| --- \| --- \| --- \| \| 2 \| icrf_period_me___2 \| Quarterly \| \| 3 \| icrf_period_me___3 \| Monthly \| \| 99 \| icrf_period_me___99 \| Other (specify in notes) \|   Custom alignment: LV |
|  | 214 | [icrf_period_re]  Show the field ONLY if:  [icrf_re]='1' |  | checkbox, Required   \| 1 \| icrf_period_re___1 \| Annual \| \| --- \| --- \| --- \| \| 2 \| icrf_period_re___2 \| Quarterly \| \| 3 \| icrf_period_re___3 \| Monthly \| \| 99 \| icrf_period_re___99 \| Other (specify in notes) \|   Custom alignment: LV |
|  | 215 | [icrf_period_ms]  Show the field ONLY if:  [icrf_ms]='1' |  | checkbox, Required   \| 1 \| icrf_period_ms___1 \| Annual \| \| --- \| --- \| --- \| \| 2 \| icrf_period_ms___2 \| Quarterly \| \| 3 \| icrf_period_ms___3 \| Monthly \| \| 99 \| icrf_period_ms___99 \| Other (specify in notes) \|   Custom alignment: LV |
|  | 216 | [icrf_period_bs]  Show the field ONLY if:  [icrf_bs]='1' |  | checkbox, Required   \| 1 \| icrf_period_bs___1 \| Annual \| \| --- \| --- \| --- \| \| 2 \| icrf_period_bs___2 \| Quarterly \| \| 3 \| icrf_period_bs___3 \| Monthly \| \| 99 \| icrf_period_bs___99 \| Other (specify in notes) \|   Custom alignment: LV |
|  | 217 | [icrf_period_abu]  Show the field ONLY if:  [icrf_abu]='1' |  | checkbox, Required   \| 1 \| icrf_period_abu___1 \| Annual \| \| --- \| --- \| --- \| \| 2 \| icrf_period_abu___2 \| Quarterly \| \| 3 \| icrf_period_abu___3 \| Monthly \| \| 99 \| icrf_period_abu___99 \| Other (specify in notes) \|   Custom alignment: LV |
|  | 218 | [icrf_period_int]  Show the field ONLY if:  [icrf_int]='1' |  | checkbox, Required   \| 1 \| icrf_period_int___1 \| Annual \| \| --- \| --- \| --- \| \| 2 \| icrf_period_int___2 \| Quarterly \| \| 3 \| icrf_period_int___3 \| Monthly \| \| 99 \| icrf_period_int___99 \| Other (specify in notes) \|   Custom alignment: LV |
|  | 219 | [icrf_period_npb]  Show the field ONLY if:  [icrf_npb]='1' |  | checkbox, Required   \| 1 \| icrf_period_npb___1 \| Annual \| \| --- \| --- \| --- \| \| 2 \| icrf_period_npb___2 \| Quarterly \| \| 3 \| icrf_period_npb___3 \| Monthly \| \| 99 \| icrf_period_npb___99 \| Other (specify in notes) \|   Custom alignment: LV |
|  | 220 | [icrf_period_plu]  Show the field ONLY if:  [icrf_plu]='1' |  | checkbox, Required   \| 1 \| icrf_period_plu___1 \| Annual \| \| --- \| --- \| --- \| \| 2 \| icrf_period_plu___2 \| Quarterly \| \| 3 \| icrf_period_plu___3 \| Monthly \| \| 99 \| icrf_period_plu___99 \| Other (specify in notes) \|   Custom alignment: LV |
|  | 221 | [icrf_geo_agg_me]  Show the field ONLY if:  [icrf_me]='1' |  | checkbox, Required   \| 1 \| icrf_geo_agg_me___1 \| National \| \| --- \| --- \| --- \| \| 2 \| icrf_geo_agg_me___2 \| Regional \| \| 3 \| icrf_geo_agg_me___3 \| State \| \| 4 \| icrf_geo_agg_me___4 \| State Region \| \| 5 \| icrf_geo_agg_me___5 \| County \| \| 6 \| icrf_geo_agg_me___6 \| Smaller than county (specify) \|   Custom alignment: LV |
|  | 222 | [icrf_geo_agg_re]  Show the field ONLY if:  [icrf_re]='1' |  | checkbox, Required   \| 1 \| icrf_geo_agg_re___1 \| National \| \| --- \| --- \| --- \| \| 2 \| icrf_geo_agg_re___2 \| Regional \| \| 3 \| icrf_geo_agg_re___3 \| State \| \| 4 \| icrf_geo_agg_re___4 \| State Region \| \| 5 \| icrf_geo_agg_re___5 \| County \| \| 6 \| icrf_geo_agg_re___6 \| Smaller than county (specify) \|   Custom alignment: LV |
|  | 223 | [icrf_geo_agg_ms]  Show the field ONLY if:  [icrf_ms]='1' |  | checkbox, Required   \| 1 \| icrf_geo_agg_ms___1 \| National \| \| --- \| --- \| --- \| \| 2 \| icrf_geo_agg_ms___2 \| Regional \| \| 3 \| icrf_geo_agg_ms___3 \| State \| \| 4 \| icrf_geo_agg_ms___4 \| State Region \| \| 5 \| icrf_geo_agg_ms___5 \| County \| \| 6 \| icrf_geo_agg_ms___6 \| Smaller than county (specify) \|   Custom alignment: LV |
|  | 224 | [icrf_geo_agg_bs]  Show the field ONLY if:  [icrf_bs]='1' |  | checkbox, Required   \| 1 \| icrf_geo_agg_bs___1 \| National \| \| --- \| --- \| --- \| \| 2 \| icrf_geo_agg_bs___2 \| Regional \| \| 3 \| icrf_geo_agg_bs___3 \| State \| \| 4 \| icrf_geo_agg_bs___4 \| State Region \| \| 5 \| icrf_geo_agg_bs___5 \| County \| \| 6 \| icrf_geo_agg_bs___6 \| Smaller than county (specify) \|   Custom alignment: LV |
|  | 225 | [icrf_geo_agg_abu]  Show the field ONLY if:  [icrf_abu]='1' |  | checkbox, Required   \| 1 \| icrf_geo_agg_abu___1 \| National \| \| --- \| --- \| --- \| \| 2 \| icrf_geo_agg_abu___2 \| Regional \| \| 3 \| icrf_geo_agg_abu___3 \| State \| \| 4 \| icrf_geo_agg_abu___4 \| State Region \| \| 5 \| icrf_geo_agg_abu___5 \| County \| \| 6 \| icrf_geo_agg_abu___6 \| Smaller than county (specify) \|   Custom alignment: LV |
|  | 226 | [icrf_geo_agg_int]  Show the field ONLY if:  [icrf_int]='1' |  | checkbox, Required   \| 1 \| icrf_geo_agg_int___1 \| National \| \| --- \| --- \| --- \| \| 2 \| icrf_geo_agg_int___2 \| Regional \| \| 3 \| icrf_geo_agg_int___3 \| State \| \| 4 \| icrf_geo_agg_int___4 \| State Region \| \| 5 \| icrf_geo_agg_int___5 \| County \| \| 6 \| icrf_geo_agg_int___6 \| Smaller than county (specify) \|   Custom alignment: LV |
|  | 227 | [icrf_geo_agg_npb]  Show the field ONLY if:  [icrf_npb]='1' |  | checkbox, Required   \| 1 \| icrf_geo_agg_npb___1 \| National \| \| --- \| --- \| --- \| \| 2 \| icrf_geo_agg_npb___2 \| Regional \| \| 3 \| icrf_geo_agg_npb___3 \| State \| \| 4 \| icrf_geo_agg_npb___4 \| State Region \| \| 5 \| icrf_geo_agg_npb___5 \| County \| \| 6 \| icrf_geo_agg_npb___6 \| Smaller than county (specify) \|   Custom alignment: LV |
|  | 228 | [icrf_geo_agg_plu]  Show the field ONLY if:  [icrf_plu]='1' |  | checkbox, Required   \| 1 \| icrf_geo_agg_plu___1 \| National \| \| --- \| --- \| --- \| \| 2 \| icrf_geo_agg_plu___2 \| Regional \| \| 3 \| icrf_geo_agg_plu___3 \| State \| \| 4 \| icrf_geo_agg_plu___4 \| State Region \| \| 5 \| icrf_geo_agg_plu___5 \| County \| \| 6 \| icrf_geo_agg_plu___6 \| Smaller than county (specify) \|   Custom alignment: LV |
|  | 229 | [icrf_notes_me]  Show the field ONLY if:  [icrf_me]='1' |  | notes |
|  | 230 | [icrf_notes_re]  Show the field ONLY if:  [icrf_re]='1' |  | notes |
|  | 231 | [icrf_notes_ms]  Show the field ONLY if:  [icrf_ms]='1' |  | notes |
|  | 232 | [icrf_notes_bs]  Show the field ONLY if:  [icrf_bs]='1' |  | notes |
|  | 233 | [icrf_notes_abu]  Show the field ONLY if:  [icrf_abu]='1' |  | notes |
|  | 234 | [icrf_notes_int]  Show the field ONLY if:  [icrf_int]='1' |  | notes |
|  | 235 | [icrf_notes_npb]  Show the field ONLY if:  [icrf_npb]='1' |  | notes |
|  | 236 | [icrf_notes_plu]  Show the field ONLY if:  [icrf_plu]='1' |  | notes |
|  | 237 | [health_system_char] | Section Header: *Section 2: Indicators and Reporting Format among Reviewed Maternal Health Dashboards D) HEALTH SYSTEM CHARACTERISTICS*  Does the dashboard have any indicators on Health System Characteristics? | yesno, Required   \| 1 \| Yes \| \| --- \| --- \| \| 0 \| No \|   Custom alignment: LV |
|  | 238 | [hsc_table]  Show the field ONLY if:  [health_system_char] = '1' | Category Any Indicator Format Time period Geography Notes Policy Measures {hsc_pm} {hsc_format_pm} {hsc_period_pm} {hsc_geo_agg_pm} {hsc_notes_pm} Availability of care {hsc_ac} {hsc_format_ac} {hsc_period_ac} {hsc_geo_agg_ac} {hsc_notes_ac} Healthcare expenditures {hsc_he} {hsc_format_he} {hsc_period_he} {hsc_geo_agg_he} {hsc_notes_he} Population characteristics {hsc_pm_2} {hsc_format_pm_2} {hsc_period_pm_2} {hsc_geo_agg_pm_2} {hsc_notes_pm_2} WIC Certified Stores {hsc_pm_3} {hsc_format_pm_3} {hsc_period_pm_3} {hsc_geo_agg_pm_3} {hsc_notes_pm_3} Other (please specify) {other_hsc_category} {other_hsc_exists} {other_hsc_format} {other_hsc_period} {other_hsc_geo_agg} {hsc_notes_other} | descriptive |
|  | 239 | [other_hsc_category]  Show the field ONLY if:  [other_hsc_exists]='1' | Other hsc category | text, Required Custom alignment: LV |
|  | 240 | [hsc_pm]  Show the field ONLY if:  [health_system_char]='1' | Policy measures data | yesno, Required   \| 1 \| Yes \| \| --- \| --- \| \| 0 \| No \|   Custom alignment: LV |
|  | 241 | [hsc_ac]  Show the field ONLY if:  [health_system_char]='1' | availability of care data | yesno, Required   \| 1 \| Yes \| \| --- \| --- \| \| 0 \| No \|   Custom alignment: LV |
|  | 242 | [hsc_he]  Show the field ONLY if:  [health_system_char]='1' | healthcare expenditures data | yesno, Required   \| 1 \| Yes \| \| --- \| --- \| \| 0 \| No \|   Custom alignment: LV |
|  | 243 | [other_hsc_exists]  Show the field ONLY if:  [health_system_char]='1' | Other hsc data | yesno, Required   \| 1 \| Yes \| \| --- \| --- \| \| 0 \| No \|   Custom alignment: LV |
|  | 244 | [hsc_format_pm]  Show the field ONLY if:  [hsc_pm]='1' | Policy measures data format | checkbox, Required   \| 1 \| hsc_format_pm___1 \| Numbers \| \| --- \| --- \| --- \| \| 2 \| hsc_format_pm___2 \| Rates/% \| \| 99 \| hsc_format_pm___99 \| Other (specify in notes) \|   Custom alignment: LV |
|  | 245 | [hsc_format_ac]  Show the field ONLY if:  [hsc_ac]='1' | Availability of care data format | checkbox, Required   \| 1 \| hsc_format_ac___1 \| Numbers \| \| --- \| --- \| --- \| \| 2 \| hsc_format_ac___2 \| Rates/% \| \| 99 \| hsc_format_ac___99 \| Other (specify in notes) \|   Custom alignment: LV |
|  | 246 | [hsc_format_he]  Show the field ONLY if:  [hsc_he]='1' | Healthcare expenditures data format | checkbox, Required   \| 1 \| hsc_format_he___1 \| Numbers \| \| --- \| --- \| --- \| \| 2 \| hsc_format_he___2 \| Rates/% \| \| 99 \| hsc_format_he___99 \| Other (specify in notes) \|   Custom alignment: LV |
|  | 247 | [other_hsc_format]  Show the field ONLY if:  [other_hsc_exists]='1' | Other HSC data format | checkbox, Required   \| 1 \| other_hsc_format___1 \| Numbers \| \| --- \| --- \| --- \| \| 2 \| other_hsc_format___2 \| Rates/% \| \| 99 \| other_hsc_format___99 \| Other (specify in notes) \|   Custom alignment: LV |
|  | 248 | [hsc_period_pm]  Show the field ONLY if:  [hsc_pm]='1' | Policy measures data time period | checkbox, Required   \| 1 \| hsc_period_pm___1 \| Annual \| \| --- \| --- \| --- \| \| 2 \| hsc_period_pm___2 \| Quarterly \| \| 3 \| hsc_period_pm___3 \| Monthly \| \| 99 \| hsc_period_pm___99 \| Other (specify in notes) \|   Custom alignment: LV |
|  | 249 | [hsc_period_ac]  Show the field ONLY if:  [hsc_ac]='1' | Availability of care data time period | checkbox, Required   \| 1 \| hsc_period_ac___1 \| Annual \| \| --- \| --- \| --- \| \| 2 \| hsc_period_ac___2 \| Quarterly \| \| 3 \| hsc_period_ac___3 \| Monthly \| \| 99 \| hsc_period_ac___99 \| Other (specify in notes) \|   Custom alignment: LV |
|  | 250 | [hsc_period_he]  Show the field ONLY if:  [hsc_he]='1' | Healthcare expenditures data time period | checkbox, Required   \| 1 \| hsc_period_he___1 \| Annual \| \| --- \| --- \| --- \| \| 2 \| hsc_period_he___2 \| Quarterly \| \| 3 \| hsc_period_he___3 \| Monthly \| \| 99 \| hsc_period_he___99 \| Other (specify in notes) \|   Custom alignment: LV |
|  | 251 | [other_hsc_period]  Show the field ONLY if:  [other_hsc_exists]='1' | Other HSC data time period | checkbox, Required   \| 1 \| other_hsc_period___1 \| Annual \| \| --- \| --- \| --- \| \| 2 \| other_hsc_period___2 \| Quarterly \| \| 3 \| other_hsc_period___3 \| Monthly \| \| 99 \| other_hsc_period___99 \| Other (specify in notes) \|   Custom alignment: LV |
|  | 252 | [hsc_geo_agg_pm]  Show the field ONLY if:  [hsc_pm]='1' | Policy measures data geographic aggregation | checkbox, Required   \| 1 \| hsc_geo_agg_pm___1 \| National \| \| --- \| --- \| --- \| \| 2 \| hsc_geo_agg_pm___2 \| Regional \| \| 3 \| hsc_geo_agg_pm___3 \| State \| \| 4 \| hsc_geo_agg_pm___4 \| State Region \| \| 5 \| hsc_geo_agg_pm___5 \| County \| \| 6 \| hsc_geo_agg_pm___6 \| Smaller than county (specify) \|   Custom alignment: LV |
|  | 253 | [hsc_geo_agg_ac]  Show the field ONLY if:  [hsc_ac]='1' | Availability of care data geographic aggregation | checkbox, Required   \| 1 \| hsc_geo_agg_ac___1 \| National \| \| --- \| --- \| --- \| \| 2 \| hsc_geo_agg_ac___2 \| Regional \| \| 3 \| hsc_geo_agg_ac___3 \| State \| \| 4 \| hsc_geo_agg_ac___4 \| State Region \| \| 5 \| hsc_geo_agg_ac___5 \| County \| \| 6 \| hsc_geo_agg_ac___6 \| Smaller than county (specify) \|   Custom alignment: LV |
|  | 254 | [hsc_geo_agg_he]  Show the field ONLY if:  [hsc_he]='1' | Healthcare expenditures data geographic aggregation | checkbox, Required   \| 1 \| hsc_geo_agg_he___1 \| National \| \| --- \| --- \| --- \| \| 2 \| hsc_geo_agg_he___2 \| Regional \| \| 3 \| hsc_geo_agg_he___3 \| State \| \| 4 \| hsc_geo_agg_he___4 \| State Region \| \| 5 \| hsc_geo_agg_he___5 \| County \| \| 6 \| hsc_geo_agg_he___6 \| Smaller than county (specify) \|   Custom alignment: LV |
|  | 255 | [other_hsc_geo_agg]  Show the field ONLY if:  [other_hsc_exists]='1' | Other HSC data geographic aggregation | checkbox, Required   \| 1 \| other_hsc_geo_agg___1 \| National \| \| --- \| --- \| --- \| \| 2 \| other_hsc_geo_agg___2 \| Regional \| \| 3 \| other_hsc_geo_agg___3 \| State \| \| 4 \| other_hsc_geo_agg___4 \| State Region \| \| 5 \| other_hsc_geo_agg___5 \| County \| \| 6 \| other_hsc_geo_agg___6 \| Smaller than county (specify) \|   Custom alignment: LV |
|  | 256 | [hsc_notes_pm]  Show the field ONLY if:  [hsc_pm]='1' | Policy measures notes | notes |
|  | 257 | [hsc_notes_ac]  Show the field ONLY if:  [hsc_ac]='1' | Availability of care notes | notes |
|  | 258 | [hsc_notes_he]  Show the field ONLY if:  [hsc_he]='1' | Healthcare expenditures notes | notes |
|  | 259 | [hsc_notes_other]  Show the field ONLY if:  [other_hsc_exists]='1' | Other HSC indicators notes | notes |
|  | 260 | [hsc_pm_2]  Show the field ONLY if:  [health_system_char]='1' | Population characteristics data | yesno, Required   \| 1 \| Yes \| \| --- \| --- \| \| 0 \| No \|   Custom alignment: LV |
|  | 261 | [hsc_pm_3]  Show the field ONLY if:  [health_system_char]='1' | WIC Stores data | yesno, Required   \| 1 \| Yes \| \| --- \| --- \| \| 0 \| No \|   Custom alignment: LV |
|  | 262 | [hsc_format_pm_2]  Show the field ONLY if:  [hsc_pm_2]='1' | Population characteristics data format | checkbox, Required   \| 1 \| hsc_format_pm_2___1 \| Numbers \| \| --- \| --- \| --- \| \| 2 \| hsc_format_pm_2___2 \| Rates/% \| \| 99 \| hsc_format_pm_2___99 \| Other (specify in notes) \|   Custom alignment: LV |
|  | 263 | [hsc_format_pm_3]  Show the field ONLY if:  [hsc_pm_3]='1' | WIC stores data format | checkbox, Required   \| 1 \| hsc_format_pm_3___1 \| Numbers \| \| --- \| --- \| --- \| \| 2 \| hsc_format_pm_3___2 \| Rates/% \| \| 99 \| hsc_format_pm_3___99 \| Other (specify in notes) \|   Custom alignment: LV |
|  | 264 | [hsc_period_pm_2]  Show the field ONLY if:  [hsc_pm_2]='1' | Population characteristics data time period | checkbox, Required   \| 1 \| hsc_period_pm_2___1 \| Annual \| \| --- \| --- \| --- \| \| 2 \| hsc_period_pm_2___2 \| Quarterly \| \| 3 \| hsc_period_pm_2___3 \| Monthly \| \| 99 \| hsc_period_pm_2___99 \| Other (specify in notes) \|   Custom alignment: LV |
|  | 265 | [hsc_period_pm_3]  Show the field ONLY if:  [hsc_pm_3]='1' | WIC stores data time period | checkbox, Required   \| 1 \| hsc_period_pm_3___1 \| Annual \| \| --- \| --- \| --- \| \| 2 \| hsc_period_pm_3___2 \| Quarterly \| \| 3 \| hsc_period_pm_3___3 \| Monthly \| \| 99 \| hsc_period_pm_3___99 \| Other (specify in notes) \|   Custom alignment: LV |
|  | 266 | [hsc_geo_agg_pm_2]  Show the field ONLY if:  [hsc_pm_2]='1' | Population characteristics data geographic aggregation | checkbox, Required   \| 1 \| hsc_geo_agg_pm_2___1 \| National \| \| --- \| --- \| --- \| \| 2 \| hsc_geo_agg_pm_2___2 \| Regional \| \| 3 \| hsc_geo_agg_pm_2___3 \| State \| \| 4 \| hsc_geo_agg_pm_2___4 \| State Region \| \| 5 \| hsc_geo_agg_pm_2___5 \| County \| \| 6 \| hsc_geo_agg_pm_2___6 \| Smaller than county (specify) \|   Custom alignment: LV |
|  | 267 | [hsc_geo_agg_pm_3]  Show the field ONLY if:  [hsc_pm_3]='1' | WIC stores data geographic aggregation | checkbox, Required   \| 1 \| hsc_geo_agg_pm_3___1 \| National \| \| --- \| --- \| --- \| \| 2 \| hsc_geo_agg_pm_3___2 \| Regional \| \| 3 \| hsc_geo_agg_pm_3___3 \| State \| \| 4 \| hsc_geo_agg_pm_3___4 \| State Region \| \| 5 \| hsc_geo_agg_pm_3___5 \| County \| \| 6 \| hsc_geo_agg_pm_3___6 \| Smaller than county (specify) \|   Custom alignment: LV |
|  | 268 | [hsc_notes_pm_2]  Show the field ONLY if:  [hsc_pm_2]='1' | Population characteristics notes | notes |
|  | 269 | [hsc_notes_pm_3]  Show the field ONLY if:  [hsc_pm_3]='1' | WIC stores notes | notes |
|  | 270 | [overall_comments] | Section Header: *Section 3: Reviewer details*  Overall Comments | notes |
|  | 271 | [initial_completer] | Initial Completer | dropdown (autocomplete), Required   \| 1 \| CB \| \| --- \| --- \| \| 2 \| GK \| \| 3 \| JC \| \| 4 \| PN \| \| 5 \| TT \| |
|  | 272 | [reviewer_1] | Reviewer 1 | dropdown (autocomplete)   \| 1 \| CB \| \| --- \| --- \| \| 2 \| GK \| \| 3 \| JC \| \| 4 \| PN \| \| 5 \| TT \| |
|  | 273 | [reviewer_2] | Reviewer 2 | dropdown (autocomplete)   \| 1 \| CB \| \| --- \| --- \| \| 2 \| GK \| \| 3 \| JC \| \| 4 \| PN \| \| 5 \| TT \| |
|  | 274 | [dashboard_features_and_indicators_complete] | Section Header: *Form Status*  Complete? | dropdown   \| 0 \| Incomplete \| \| --- \| --- \| \| 1 \| Unverified \| \| 2 \| Complete \| |
