# Supplementary material for: Public Maternal Health Dashboards in the United States: Descriptive Assessment
Source: J Med Internet Res. 2024 Sep 17;26:e56804. doi: 10.2196/56804 (PMC11445621; doi:10.2196/56804)
Supplement: Multimedia Appendix 3 [file jmir_v26i1e56804_app3.docx]

**Supplemental File 3. Description of Other Categories for Tables 1, 3, and 4**

Table 1: Design and Features of Reviewed Maternal Health Dashboards

**Hosting sites, other types observed:** ArcGIS; Coalition/Public Private Partnership website; County/city website; Federal Agency website; HRSA website; State data center (run by state library); State Kids Count website; UNICEF website; University (research center) websites.

**Data visualizations, other types observed:** Area charts; Donut charts; Large text; Pictogram; Pyramid charts; Radial chart; Stacked bar charts.

**Technical capabilities, other types observed:** Animated charts; Color-blind mode; Create slide stack of specific visualizations; Dark mode; Detail adjustment knob; Drag to select; Focus mode; Page printing; Sharing links.

**Audience type, other types observed:** Businesses; Chambers of Commerce; Journalists/media; Libraries/medical librarians; Master planners; Not-for-profit organizations; Professional societies/health associations; Schools; Students; Teachers; Tourists; Voluntary agencies.

Table 3. Data Sources, Comparisons, and Disaggregation Capabilities among Reviewed Maternal Health Dashboards

**Sources of data, other types stated on dashboards:** Annie E. Casey Foundation Kids Count Data Center/State Kids Count; Area-level population data; Behavioral Risk Factor Surveillance System; California Maternal and Infant Health Assessment (MIHA); Centers for Disease Control and Prevention (CDC) (non-specific); CDC Breastfeeding report card; Claritas Consumer Profiles; CMS Hospital Compare; Common Formats for Event Reporting - Hospital Version 1.2 (CFER-H V1.2); Consumer Assessment of Healthcare Providers and Systems (CAHPS); County Health Rankings; ER Visits data; Guttmacher Institute; Healthcare Cost and Utilization Project (HCUP); Health Plan (Medicaid) Administrative Data; Healthcare Effectiveness Data and Information Set (HEDIS); Illinois Department of Public Health, Public Data; Illinois Public Health Community Map; Integrated Public Use Microdata Series (IPUMS-USA); Kaiser Family Foundation; Maine Office of Children and Family Services; March of Dimes; Massachusetts Population Health Information Tool; Maternal, Infant, and Early Childhood Home Visiting Statewide Needs Assessment; Motor vehicle crash data; National Birth Defects Prevention Network; National Immunization Survey; National Survey of Child's Health; New Hampshire Pregnancy Nutrition Surveillance System; New Mexico Indicator-Based Information System; National Plan and Provider Enumeration System, National Provider Identifier (NPI) records; Oregon Public Health Assessment Tool (OPHAT); Statewide Planning and Research Cooperative System; State of California Department of Finance; Statewide Medicaid Data; STD data; Title V Federally Available Data; UNICEF "Direct Communications”; UNICEF Multiple Indicator Cluster Survey (MICS); USDA Food Environment Atlas; Youth Risk Behavior Surveillance System.

**Disaggregation capabilities, other types observed:** Access to contraception; Birth spacing; Breastfeeding; Cause of pregnancy-associated maternal death; Cause of infant death; Disability status; Language spoken; Maternal BMI; Method of delivery; Number of live births; Number of premature births; Parity (counting stillbirths - any pregnancy that was carried to term); Performance quartile (on metric); Perinatal level of care (I-III+); Physical abuse; Population density; Pregnancy intention; Preventability of pregnancy-associated maternal death; Received first trimester prenatal care; Timing of infant death; Timing of pregnancy-associated maternal death; Use of contraception.

Table 4. Variables

**Health status, other variables observed:** Assisted Ventilation for 6+ hours; Births occurring in baby friendly facilities; Cervical Dilation at admission (less than 3cm, 4cm, or more); Epidural or spinal anesthesia; Episiotomy; Extent of harm (from c-section); Fetal deaths/late fetal mortality; Immediate assisted ventilation; Induction (Yes/No); Maternal Asthma; Maternal COVID-19; Maternal heart problems; Maternal infections, bacterial; Maternal infections, STI/STD; Maternal thyroid problems; Maternal/postpartum anemia; Median 5-min APGAR score; NICU admission; Obstetric hemorrhage; Use of antibiotics; Women who gave birth diagnosed with opioid use disorder per 1000 delivery hospitalizations.

**Health behaviors and utilization, other variables observed:** Abortion/induced pregnancy terminations; Abortion-related complications; Attendant at birth; Care-seeking for fever; Chlamydia screening in women; Contraceptive use/method/effectiveness; Early elective induction; Fertility treatment; Home visits; Households that received maternity care services; Initiation of injectable progesterone for preterm birth prevention; Knowledge of shaken baby syndrome; Medicaid reimbursement; Percent of infants with neonatal abstinence syndrome (NAS) enrolled to an early intervention program within 1 year after birth; Percent of infants with NAS readmitted to a hospital; Percent of infants with NAS referred to an early intervention program within six months after birth; Percent of clients who receive reproductive healthcare services through department of public health family planning programs; Percentage of pregnant participants in the Special Supplemental Nutrition Program for Women, Infants, and Children (WIC) who are co-enrolled in Medicaid; Percent of women served at WIC sites who enrolled in the WIC Program during their first trimester of pregnancy; Place of birth; Postpartum visit barriers; Percent of pregnant women who attended a childbirth class; Provider screening/discussion for IPV before/during/after pregnancy; Weight gain counseling; WIC enrollment.

**Individual characteristics and risk factors, other variables observed:** Availability of prenatal care; Nativity; Prior poor pregnancy outcomes, live births; Prior poor pregnancy outcomes, mortality; Sexual activity.

**Health system characteristics, other variables observed:** Childhood vaccination rates; Health network regions; Prenatal periods of risk.
