# Supplementary material for: Public Maternal Health Dashboards in the United States: Descriptive Assessment
Source: J Med Internet Res. 2024 Sep 17;26:e56804. doi: 10.2196/56804 (PMC11445621; doi:10.2196/56804)
Supplement: Multimedia Appendix 4 [file jmir_v26i1e56804_app4.docx]

**Supplemental File 4. Indicator Reporting Time Periods**

| Category |  |  | Time Period^1^ | | | |
| --- | --- | --- | --- | --- | --- | --- |
|  | Any Indicator |  | Annual | Quarterly | Monthly | Multiyear/ Other^2^ |
| **Health status** |  |  |  |  |  |  |
| Birthweight | 54 (68%) |  | 41 (51%) | 1 (1%) | 0 (0%) | 14 (18%) |
| Preterm birth | 45 (56%) |  | 38 (48%) | 1 (1%) | 0 (0%) | 8 (10%) |
| Infant mortality | 42 (53%) |  | 33 (41%) | 0 (0%) | 0 (0%) | 12 (15%) |
| Births | 38 (48%) |  | 30 (38%) | 1 (1%) | 2 (3%) | 11 (14%) |
| Mode of delivery | 21 (26%) |  | 20 (25%) | 1 (1%) | 0 (0%) | 1 (1%) |
| Pregnancy-related problems | 16 (20%) |  | 13 (16%) | 1 (1%) | 0 (0%) | 2 (3%) |
| Maternal depression/anxiety | 14 (18%) |  | 11 (14%) | 0 (0%) | 0 (0%) | 4 (5%) |
| Severe maternal morbidity | 12 (15%) |  | 10 (13%) | 1 (1%) | 0 (0%) | 1 (1%) |
| Maternal mortality | 10 (13%) |  | 7 (9%) | 0 (0%) | 0 (0%) | 4 (5%) |
| Birth defects | 9 (11%) |  | 7 (9%) | 0 (0%) | 0 (0%) | 3 (4%) |
| Neonatal abstinence syndrome | 8 (10%) |  | 7 (9%) | 0 (0%) | 0 (0%) | 3 (4%) |
| Other | 14 (18%) |  | 14 (18%) | 0 (0%) | 0 (0%) | 2 (3%) |
| **Health behaviors and healthcare utilization** | | | | | | |
| Prenatal care | 57 (71%) |  | 47 (59%) | 0 (0%) | 1 (1%) | 12 (15%) |
| Maternal smoking | 42 (53%) |  | 35 (44%) | 0 (0%) | 1 (1%) | 9 (11%) |
| Breastfeeding | 22 (28%) |  | 19 (24%) | 0 (0%) | 0 (0%) | 3 (4%) |
| Substance use | 14 (18%) |  | 13 (16%) | 0 (0%) | 0 (0%) | 2 (3%) |
| Oral health | 13 (16%) |  | 12 (15%) | 0 (0%) | 0 (0%) | 1 (1%) |
| Postpartum care | 12 (15%) |  | 9 (11%) | 0 (0%) | 0 (0%) | 4 (5%) |
| Maternal nutrition | 11 (14%) |  | 8 (10%) | 0 (0%) | 0 (0%) | 3 (4%) |
| Immunization | 10 (13%) |  | 9 (11%) | 0 (0%) | 0 (0%) | 1 (1%) |
| Contraception | 9 (11%) |  | 9 (11%) | 0 (0%) | 0 (0%) | 1 (1%) |
| Preventative visit | 5 (6%) |  | 5 (6%) | 0 (0%) | 0 (0%) | 0 (0%) |
| Smoking household | 5 (6%) |  | 5 (6%) | 0 (0%) | 0 (0%) | 0 (0%) |
| Other | 19 (24%) |  | 19 (24%) | 0 (0%) | 0 (0%) | 2 (3%) |
| **Individual characteristics and risk factors** | | | | | | |
| Maternal age | 43 (54%) |  | 32 (40%) | 1 (1%) | 0 (0%) | 10 (13%) |
| Preconception health | 18 (23%) |  | 15 (19%) | 0 (0%) | 0 (0%) | 3 (4%) |
| Maternal BMI | 17 (21%) |  | 15 (19%) | 0 (0%) | 1 (1%) | 2 (3%) |
| Health insurance status | 17 (21%) |  | 15 (19%) | 0 (0%) | 1 (1%) | 2 (3%) |
| Maternal education | 17 (21%) |  | 14 (18%) | 0 (0%) | 1 (1%) | 3 (4%) |
| Pregnancy intention | 13 (16%) |  | 12 (15%) | 0 (0%) | 0 (0%) | 1 (1%) |
| Stress/abuse | 11 (14%) |  | 9 (11%) | 0 (0%) | 0 (0%) | 2 (3%) |
| Maternal marital status | 10 (13%) |  | 9 (11%) | 0 (0%) | 1 (1%) | 1 (1%) |
| Plurality | 9 (11%) |  | 9 (11%) | 0 (0%) | 1 (1%) | 0 (0%) |
| Birth spacing | 7 (9%) |  | 6 (8%) | 0 (0%) | 1 (1%) | 1 (1%) |
| Number of prior births | 7 (9%) |  | 7 (9%) | 0 (0%) | 1 (1%) | 0 (0%) |
| Maternal race/ethnicity | 5 (6%) |  | 3 (4%) | 1 (1%) | 0 (0%) | 1 (1%) |
| Other | 2 (3%) |  | 2 (3%) | 0 (0%) | 0 (0%) | 0 (0%) |
| **Health system characteristics** | | | | | | |
| Population characteristics | 18 (23%) |  | 17 (21%) | 0 (0%) | 1 (1%) | 4 (5%) |
| WIC access | 16 (20%) |  | 10 (13%) | 0 (0%) | 1 (1%) | 5 (6%) |
| Availability of maternity care | 12 (15%) |  | 7 (9%) | 1 (1%) | 0 (0%) | 6 (8%) |
| Policy measures | 4 (5%) |  | 3 (4%) | 0 (0%) | 0 (0%) | 1 (1%) |
| Healthcare expenditures | 2 (3%) |  | 2 (3%) | 0 (0%) | 0 (0%) | 0 (0%) |
| Other | 3 (4%) |  | 2 (3%) | 0 (0%) | 0 (0%) | 1 (1%) |

Notes: ^1^The denominator for each percentage reported in this table is 80 dashboards. Dashboards may use more than one reporting format and more than one geographic level.^2^ The most common “other” time period was multiyear periods, such as two to three years combined. Variables reported for only one time period (e.g., a single year), or unspecified time period, are also included in “other.” Acronyms: BMI=body mass index; WIC=Special Supplemental Nutrition Program for Women, Infants, and Children.
